# Asymmetric rotations and dimerization driven by normal to modulated phase transition in 4-biphenylcarboxy coupled l-phenylalaninate

**DOI:** 10.1107/S2052520623000215

**Published:** 2023-03-02

**Authors:** Somnath Dey, Supriya Sasmal, Saikat Mondal, Santosh Kumar, Rituparno Chowdhury, Debashrita Sarkar, C. Malla Reddy, Lars Peters, Georg Roth, Debasish Haldar

**Affiliations:** a Department of Chemical Sciences, Indian Institute of Science Education and Research (IISER) Kolkata, Mohanpur 741246, India; b Institute of Crystallography, RWTH Aachen University, Jägerstraße 17-19, 52066 Aachen, Germany; Edinburgh, United Kingdom

**Keywords:** phase transitions, intermolecular interactions, hydrogen bonding, molecular crystals, couple, rotations, modulation, steric

## Abstract

Amongst the derivatives of 4-biphenylcarboxylic acid and amino acid esters, the crystal structure of 4-biphenylcarboxy-(l)-phenylalaninate is unusual owing to its monoclinic symmetry within a pseudo-orthorhombic crystal system. The distortion is described by a disparate rotational property around the chiral centers (φ_chiral_ ≃ −129° and 58°) of the two molecules in the asymmetric unit. Each of these molecules comprises planar biphenyl moieties (φ_biphenyl_ = 0°). Using temperature-dependent single-crystal X-ray diffraction experiments we show that the compound undergoes a phase transition below *T* ∼ 124 K that is characterized by a commensurate modulation wavevector, **q** = δ(101), δ = ½. The (3+1)-dimensional modulated structure at *T* = 100 K suggests that the phase transition drives the biphenyl moieties towards noncoplanar conformations with significant variation of internal torsion angle (φ^max^_biphenyl_ ≤ 20°). These intramolecular rotations lead to dimerization of the molecular stacks that are described predominantly by distortions in intermolecular tilts (θ_max_ ≤ 20°) and small variations in intermolecular distances (Δ*d*_max_ ≃ 0.05 Å) between biphenyl molecules. Atypical of modulated structures and superstructures of biphenyl and other polyphenyls, the rotations of individual molecules are asymmetric (Δφ_biphenyl_ ≈ 5°) while φ_biphenyl_ of one independent molecule is two to four times larger than the other. Crystal-chemical analysis and phase relations in superspace suggest multiple competing factors involving intramolecular steric factors, intermolecular H—C⋯C—H contacts and weak C—H⋯O hydrogen bonds that govern the distinctively unequal torsional properties of the molecules.

## Abstract 2

Crystals of a coupled molecule of 4-biphenylcarboxy-(l)-phenylalaninate undergo normal to commensurately modulated phase transition at *T*_c_ ∼ 124 K that is characterized by torsional modulation typical of modulated structures and superstructures of polyphenyls yet unusual owing to asymmetric and unequal rotations governed by intramolecular and intermolecular constraints. The transition is presumably governed by significant suppression of atomic displacement parameters correlated to the evolution of amplitude of atomic modulation functions.

## 1. Introduction

Molecular biphenyl has been investigated extensively for its stability and conformation in different thermodynamic states. At ambient conditions, the differences in the conjugation states of the π electrons are governed primarily by the twist about the central C—C single bond in the order 40°–45° in gas phase, 20°–25° in solution and 0° (mutually coplanar) in solid state in centrosymmetric monoclinic space group *P*2_1_/*a* (Bastiansen, 1949 ▸; Suzuki, 1959 ▸; Trotter, 1961 ▸; Hargreaves & Rizvi, 1962 ▸).

The planar conformation due to constraints from intermolecular interactions is energetically unfavorable and steric hindrance between the *ortho* hydrogen atoms is compensated for by out-of-plane dynamic disorder and in-plane displacements of those hydrogen atoms away from each other (Hargreaves & Rizvi, 1962 ▸; Casalone *et al.*, 1968 ▸; Charbonneau & Delugeard, 1976 ▸; Charbonneau & Delugeard, 1977 ▸; Busing, 1983 ▸; Lenstra *et al.*, 1994 ▸). A recent study has also suggested the role of intramolecular exchange energy between single-bonded carbon atoms in stabilizing the planar conformation (Popelier *et al.*, 2019 ▸).

Absorption and fluorescence studies showed additional bands in their spectra at low temperatures (Hochstrasser *et al.*, 1973 ▸; Wakayama, 1981 ▸).

Temperature-dependent Raman spectroscopy and Brillouin scattering experiments both suggested two phase transitions at *T*_c1_ = 42 K and *T*_c2_ = 17 K (Friedman *et al.*, 1974 ▸; Bree & Edelson, 1977 ▸, 1978 ▸; Ecolivet *et al.*, 1983 ▸). The phase transition at *T*_c1_ is continuous and governed by a soft mode associated with the torsion about the central C—C single bond followed by discontinuous changes at *T*_c2_.

Inelastic neutron scattering experiments on its deuterated form confirmed the phase transitions with the appearance of additional satellite reflections (Cailleau *et al.*, 1979 ▸). The modulation wavevector **q** was determined to be **q**_I_ = 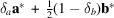 and **q**_II_ = 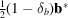 at the intermediate- and low-temperature phases, respectively. The wavevectors were found to vary with temperature, suggesting the incommensurate nature of the modulation (Cailleau *et al.*, 1979 ▸).

The modulated structure of low-temperature phase II was described within a noncentrosymmetric superspace group *Pa*(0σ_2_0)0 (de Wolff, 1974 ▸; Stokes *et al.*, 2011 ▸; van Smaalen *et al.*, 2013 ▸) and found to be essentially associated with a small modulation of translation and a rotation (ω) normal to the mean molecular plane, and a significant torsion angle (φ) between the phenyl rings (Baudour & Sanquer, 1983 ▸; Petricek *et al.*, 1985 ▸; Pinheiro & Abakumov, 2015 ▸; Schoenleber, 2011 ▸).

Theoretical studies have suggested that competition between intramolecular and intermolecular forces drives the phase transition towards the incommensurately modulated states (Ishibashi, 1981 ▸; Benkert *et al.*, 1987 ▸; Benkert & Heine, 1987 ▸; Parlinski *et al.*, 1989 ▸).

The fundamental property of flexibility in conformations has made biphenyl an excellent candidate to tune multifaceted properties in materials.

Twisting between the rings has been demonstrated to regulate conductivity of single molecule biphenyl–dithiol junctions (Vonlanthen *et al.*, 2009 ▸; Mishchenko *et al.*, 2010 ▸; Bürkle *et al.*, 2012 ▸; Jeong *et al.*, 2020 ▸), tune thermopower as a function of the twist angle (Bürkle *et al.*, 2012 ▸), degeneracy of energy states on substrates (Cranney *et al.*, 2007 ▸) and theoretically suggest wide band gap semiconducting properties of its derivatives (Khatua *et al.*, 2020 ▸). On the other hand, biphenyl derivatives have also been reported to influence and increase the efficiency of photophysical properties (Oniwa *et al.*, 2013 ▸; Wei *et al.*, 2016 ▸).

Planar biphenyl molecules in the solid state favor maximum intramolecular conjugation of π electrons as well as increasing the probability of interactions between delocalized electrons that could aid in optimal stacking of molecules.

A coupling reaction mechanism (Seechurn *et al.*, 2012 ▸) was successfully employed to synthesize 4-biphenylcarboxy protected amino acid esters of l-serine, l-tyrosine, l-alanine, l-leucine and l-phenylalanine *via* the formation of peptide-type linker O=C—NH groups (Sasmal *et al.*, 2019*b* ▸,*a* ▸).

In the solid state, the compounds crystallize either in noncentrosymmetric space group *P*2_1_2_1_2_1_ or the monoclinic subgroup *P*2_1_ (Sasmal *et al.*, 2019*b* ▸,*a* ▸). Crystal packing in these systems is determined by π⋯π stacking between the biphenyl fragments and strong linear hydrogen bonds between the amino acid ester moieties.

We presumed that the biphenyl moieties in these chemically coupled systems could influence the bioactive amino acid esters and *vice versa* with respect to evolution or suppression of translational and rotational degrees of freedom in their crystal structures at some thermodynamic condition.

**Figure 1:**
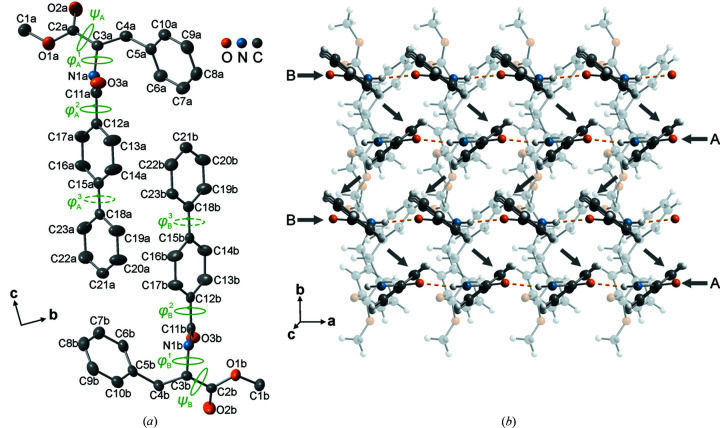
(*a*) Two independent formula units of 4-biphenylcarboxy-(l)-phenylalaninate (C_23_H_21_NO_3_) with atomic labels of non-hydrogen atoms (a and b for molecule *A* and *B*, respectively) in phase I at *T* = 160 K. 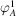 = −130.1°, 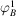 = 56.1°; 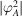 = 32.8°, 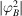 = 30.8°; 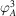 = 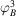 = 0°; ψ_*A*_ = 36.4°, ψ_*B*_ = 36.2°. Viewing direction along 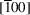 . (*b*) Crystal packing in phase I viewed along [111] emphasizing biphenyl stacks (*AA*)_*n*_ and (*BB*)_*n*_ along *a* (horizontal arrows), (*ABAB*)_*n*_ along *b* (zigzag vertical arrows) and intermolecular N—H⋯O bonds between amide groups (dashed orange) along [∓100] directions. Phenyl rings of the ester groups (transparent) are stacked only along *a*.

Reanalyzing all their crystal structures, the system of 4-biphenylcarboxy-(l)-phenylalaninate attracted our attention because the structure appeared to be similar to the l-tyrosine analog albeit the monoclinic distortion [Table 1 ▸, Sasmal *et al.* (2019*a* ▸)] and two crystallographically-independent formula units [*Z*′ = 2 (Steed & Steed, 2015 ▸), Fig. 1 ▸(*a*)] in the crystal structure of the former.

The torsion angle about the chiral center is significantly different for the independent molecules while the remainder of the rotations are similar [Fig. 1 ▸(*a*), (Sasmal *et al.*, 2019*a* ▸)].

Each of these molecules consists of coplanar biphenyl moieties which are stacked along *a* and *b*, while the amide groups are connected by intermolecular N—H⋯O hydrogen bonds [Fig. 1 ▸(*b*), Sasmal *et al.* (2019*a* ▸)].

In the present study, the temperature-dependent phase transition of 4-biphenylcarboxy-(l)-phenylalaninate has been investigated using single-crystal X-ray diffraction experiments. Low-temperature phase II is found to be a 2*a* × *b* × 2*c* superstructure of the high-temperature (phase I) structure.

The superstructure is described within the (3 + 1)D-superspace approach as a commensurately modulated structure (de Wolff, 1974 ▸; Janner & Janssen, 1977 ▸; Wagner & Schönleber, 2009 ▸; van Smaalen, 2012 ▸; Janssen *et al.*, 2018 ▸).

Structural properties of phase I and the modulated structure have been tabulated and compiled within *t*-plots (*t* = phase of the modulation). The origin and stability of phase II is discussed in terms of intramolecular steric factors and intermolecular HC⋯CH contacts and intermolecular hydrogen bonds. It is suggested that the order parameter of the phase transition is correlated with the suppression of dynamic disorder.

## 2. Experimental

### 2.1. Temperature-dependent single-crystal X-ray diffraction

Single crystals of the compound used in this study were obtained from those reported in Sasmal *et al.* (2019*a* ▸). The crystals were protected in oil under mild refrigeration. Single-crystal X-ray diffraction (SCXRD) experiments were performed on an Agilent SuperNova, Eos diffractometer employing Cu *K*α radiation. The temperature of the crystal was maintained by an Oxford Cryosystems open flow nitrogen cryostat.

During cooling, visual inspection of diffraction images revealed weaker reflections in addition to strong reflections at low temperatures.

Diffraction images collected at 150 K, 140 K and 130 K–114 K in steps of Δ*T* = 2 K showed that the weaker diffuse features appear at 124 K and condense into satellite reflections at 122 K (Table 1 ▸ and Fig. S1 in supporting information). The transition temperature is significantly higher than that of molecular biphenyl (*T*_c, biphenyl_ = 42 K). On the other hand, related polyphenyls *p*-terphenyl and *p*-quarterphenyl undergo phase transition towards superstructure phases at much higher critical temperatures [*T*_c, terphenyl_ ≈ 190 K (Yamamura *et al.*, 1998 ▸), *T*_c, quarterphenyl_ ≈ 233 K (Saito *et al.*, 1985 ▸)]. Complete diffraction data were collected at *T* = 160 K and 100 K.

Determination of unit-cell parameters and data reductions were performed using the software suite *CrysAlisPro* (Rigaku Oxford Diffraction, 2019 ▸) (Tables 1 ▸ and S1).

Satellite reflections of first order (*m* = 1) observed below *T*_c_ could be indexed with modulation wavevector **q** = (σ_1_, 0, σ_3_), σ_1_ = σ_3_ ≃ 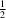 with respect to the basic monoclinic crystal system. Here, **q** = 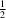 (101) is perpendicular to the *b* axis consistent with monoclinic symmetry while in molecular biphenyl **q**_I_ violates monoclinic symmetry and **q**_II_ is parallel to *b* (Cailleau *et al.*, 1979 ▸).

Using the plugin program *NADA* (Schönleber *et al.*, 2001 ▸) in *CrysAlisPro*, deviations of the σ values as a function of *T* from a rational value of 0.5 were found to be within their standard uncertainities (Table 1 ▸), indicating a commensurate nature of the modulation.

Reflections at *T* = 100 K were indexed by four integers (*hklm*) using a basic monoclinic *b*-unique lattice (Tables 1 ▸ and S1) and modulation wavevector, **q** = ( 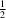 , 0, 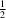 ) and data integration was performed. Empirical absorption correction was performed using the *AbsPack* program embedded in *CrysAlisPro*.

The ratio of the average intensities (〈*I*〉) between main and satellite reflections is 13:1 and that of their average significance [〈*I*/σ(*I*)〉] is 3:1. This indicates pronounced modulation which is characteristic of modulated molecular crystals (Schönleber & Chapuis, 2001 ▸; Schönleber *et al.*, 2003 ▸; Dey *et al.*, 2016 ▸, 2018 ▸; Rekis *et al.*, 2020 ▸, 2021 ▸).

The monoclinic crystal system in addition to the reflection conditions suggest the superspace group *P*2_1_(σ_1_0σ_3_)0 with σ_1_ = σ_3_ = ½ (Stokes *et al.*, 2011 ▸; van Smaalen *et al.*, 2013 ▸).

### 2.2. Structure refinement of the modulated structure

The crystal structure of the room-temperature phase (phase I hereon) was redetermined at 160 K using *Superflip* (Palatinus & Chapuis, 2007 ▸) and refined using *Jana2006* and *Jana2020* (Petříček *et al.*, 2014 ▸).

Atoms were renamed with suffixes a and b for the two independent molecules *A* and *B* [Fig. 1 ▸(*a*)]. Anisotropic atomic displacement parameters (ADPs) of all non-hydrogen atoms were refined. Hydrogen atoms were added to carbon and nitrogen atoms using a riding model in ideal chemical geometry with constraints for isotropic ADPs [*U*_iso_(H) = 1.2*U*_eq_(N), *U*_iso_(H) = 1.2*U*_eq_(C_aromatic_) and *U*_iso_(H) = 1.5*U*_eq_(C_*sp*3_)].

Owing to the pseudoorthorhombic crystal system, the integrated data was tested for twinning employing twofold rotation along the [100] direction as twin law. This twin law is a true symmetry element in the case of a hypothetical ortho­rhombic crystal system with point group symmetry 222 (Petříček *et al.*, 2016 ▸; Nespolo, 2019 ▸). The fit of the structure model improved (compare 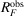 = 0.0463 to 0.0408) and volume of the second component refined to 0.0240 (8) (Table S2). Finally, positions of the H atoms of NH groups and the parameter corresponding to isotropic extinction correction were refined that further improved 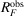 values ( 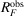 = 0.0393, Table S2 in supporting information).

The crystal structure reproduced the values for intramolecular rotations reported those for the structure at *T* = 200 K [φ_chiral_ = φ_1_ (hereon) and ψ in Fig. 1 ▸(*a*)]. In addition, we also observe that the coplanar biphenyl rings are significantly rotated with respect to the amide groups [at *T* = 200 K: φ_2_ = 32.8° and 31.2° (Sasmal *et al.*, 2019*a* ▸) and at *T* = 160 K in Fig. 1 ▸(*a*)] which also remains invariant as a function of temperature.

The modulated structure of phase II at 100 K was refined using *Jana2006* and *Jana2020*. Fractional coordinates of all atoms from the crystal structure at *T* = 160 K were used in the starting model while retaining the same riding model geometry for hydrogen atoms as in phase I and the average structure was refined as main reflections. In successive steps, an incommensurate (IC) model described by one harmonic wave for displacive modulation describing the atomic modulation functions (AMFs) and basic parameters for anisotropic ADPs for non-hydrogen atoms was refined against main and satellite reflections that resulted in good fit to the diffraction pattern ( 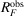 = 0.0425). However, ADPs of four non-hydrogen atoms were found to be non-positive definite.

Since the components of **q** (σ_1_ and σ_3_) are rational, three commensurately modulated structures were pursued by fixing the initial phase of the modulation to values *t*_0_ = 0, 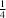 and 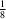 , respectively. While the former two *t*_0_ values describe monoclinic space group *B*2_1_ symmetry for the equivalent 3D 2*a* × *b* × 2*c* superstructure, the third corresponds to triclinic *B*1 symmetry. The commensurately modulated structure (C) model corresponding to *t*_0_ = 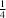 resulted in the best fit to the diffraction data ( 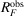 = 0.0426) including ADPs of all atoms positive definite.

**Figure 2:**
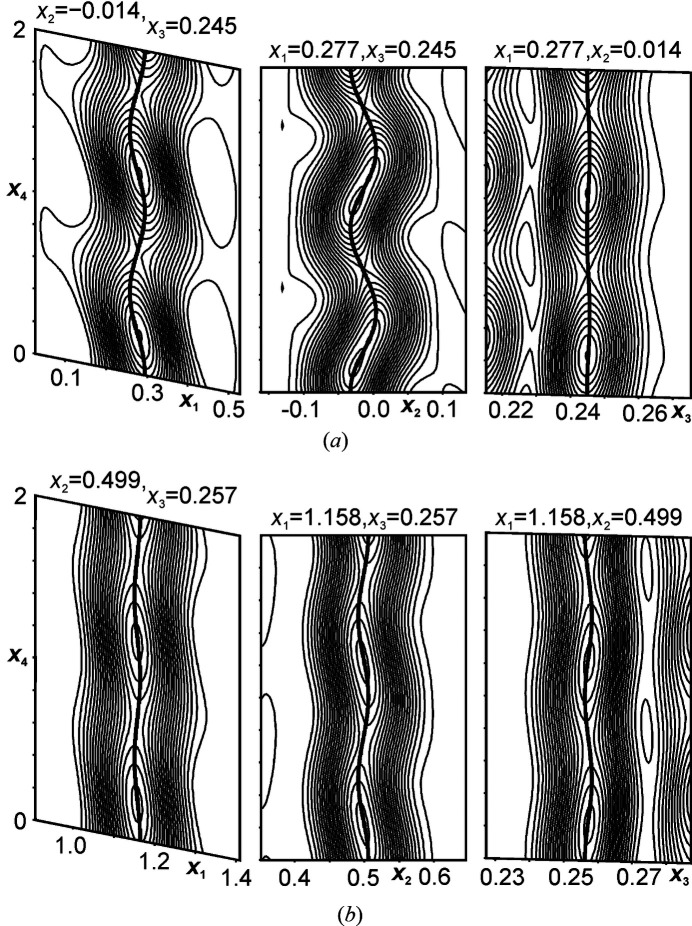
(*x*_*s*1_,*x*_*s*4_)-sections of a Fourier map centered on carbon atoms (black): (*a*) C23*a* of molecule *A* and (*b*) C23*b* of molecule *B*. The contour lines and the width of the maps are 0.5 e Å^−3^ and 2.5 Å, respectively.

As the atomic modulation functions (AMFs) have sinusoidal character, the residual values are similar to the IC model (Fig. 2 ▸, Figs. S2–S4 and Table S2 in supporting information). However, the C model at *t*_0_ = 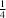 is described with either cosine or sine waves for the AMFs (equal to number of refinable fractional coordinates in the equivalent superstructure) reducing significantly the number of refinable parameters as compared with the IC model (compare *N*_C_ = 649 with *N*_IC_ = 811, further tests in supporting information).

The final C model was further improved by refining the parameter corresponding to isotropic extinction correction and AMFs and positions of hydrogen atoms of NH groups ( 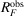 = 0.0419, Table S2). The refined twin volume in phase II reproduced the value similar to that in phase I [*T* = 100 K, twvol2 = 0.0242 (7) in Table S2]. Presumably, the crystal possesses pseudo-merohedral growth twins.

## 3. Results and discussion

### 3.1. Structural phase transition and unequal distortion of molecules

In the present case, the monoclinic symmetry is retained below *T*_c_ unlike monoclinic to triclinic distortion at the disorder–order phase transition of *p*-terphenyl (Rice *et al.*, 2013 ▸) and *p*-quarterphenyl (Baudour *et al.*, 1978 ▸).

**Figure 3:**
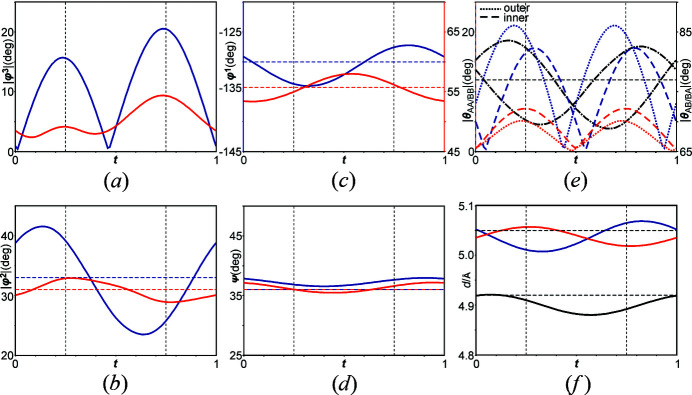
*t*-plots of intramolecular rotations of molecule *A* (blue) and *B* (red) as well as intermolecular tilts and distances between stacks of 4-biphenylcarboxy-(l)-phenylalaninate. (*a*) Dihedral angle |φ^3^| represents internal torsion within the biphenyl rings, (*b*) dihedral angle |φ^2^| represents the torsion angle between the inner ring of the biphenyl and the amide groups, (*c*) φ^1^ represents the torsion angle of the amide groups with respect to the –COOCH_3_ groups and (*d*) ψ represents the torsion angle of –COOCH_3_ groups with respect to amide groups. (*e*) | θ_*AA*/*BB*_| represents tilt between biphenyl rings of *A* and *A* ^ii^ (blue), and of *B* and *B* ^ii^ (red) and |θ_AB/BA_| (dashed-dotted black curve) represent tilt between inner aromatic rings of biphenyl (bonded to amide groups) of *A* and outer ring of *B* and *vice versa*. (*f*) Intermolecular distances (*d*) between biphenyl rings of *A* and *A* ^ii^ (blue), between those of *B* and *B* ^ii^ (red) and between those of *A* and *B* (black). Horizontal dashed lines represent those angles and distances in phase I (|φ^3^| = | θ_*AA*/*BB*_| = 0°). Vertical dashed lines indicate *t* values corresponding to angles and distances in the 3D superstructure. Symmetry code (ii): *x* + 1, *y*, *z*, *t*.

**Figure 4:**
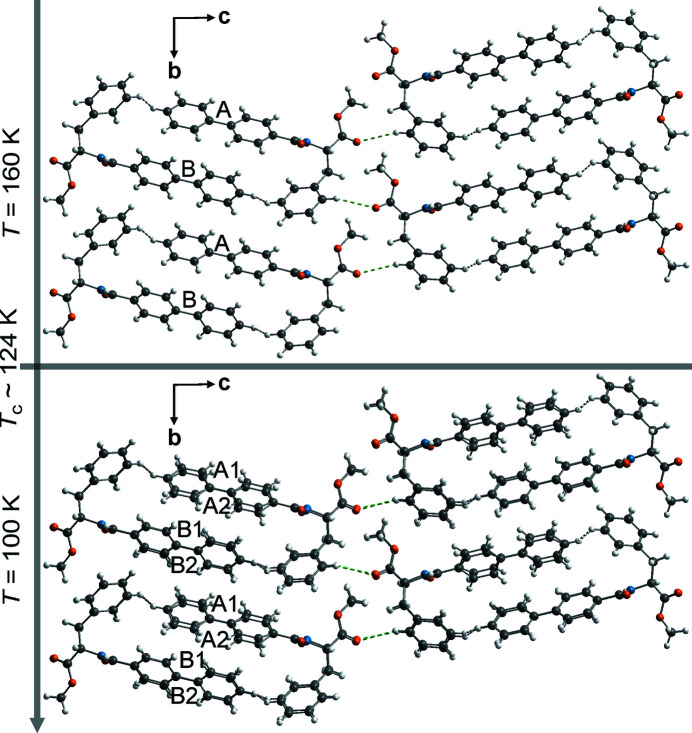
Comparison of structures in phase I and phase II across the phase transition highlighting the effect of internal torsion (φ^3^) within biphenyl on the stacking arrangements along *a*. The tilt between the biphenyl stacks, θ_*AA*/*BB*_ are different for the inner rings (bonded to amide rings) and the outer rings. Corresponding values of φ^3^ and θ are in Fig. 3 ▸(*e*) and those for C—H⋯O and C—H⋯H—C in Table 2 ▸. See full unit cells in Figs. S6 and S7. Viewed along 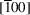 .

In the final commensurately modulated structure model with *t*_0_ = 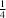 , sections corresponding to *t* = 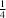 and 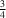 (Figs. 3 ▸ and S5) are physically relevant. These sections represent the atomic coordinates in the equivalent twofold superstructure in 3D (Figs. 4 ▸, S6 and S7).

Crystal structures of phase I and phase II have group–subgroup relations and the doubling of the *a* and *c* axes describes the additional *B*-centering of the superstructure in phase II.

The superstructure derived using *Jana2006* comprises four molecules in the asymmetric unit (*Z*′ = 4); two each corresponding to molecules *A* and *B* of phase I (Fig. 4 ▸).

The covalent bond distances are similar for the independent set of molecules and are practically unaffected by modulation Table S7).

In the present study, discussion is based on the modulated structure in order to establish unique relations between phase I and phase II (Rekis *et al.*, 2021 ▸; Chapuis, 2020 ▸; Ramakrishnan *et al.*, 2019 ▸; Dey *et al.*, 2016 ▸; Noohinejad *et al.*, 2015 ▸; Schoenleber, 2011 ▸; Schönleber *et al.*, 2003 ▸).

The modulated structure suggests that the phase transition is dominated by evolution of internal torsional degrees of freedom (φ^3^ > 0°) within the biphenyl moieties [Fig. 3 ▸(*a*)]. The twists about the central C—C bond are significantly different for the two molecules where the torsional modulation of *A* are 2–4 times larger than those of *B* (dihedral angles 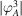 = 15.6°, 20.5° and 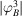 = 4.1°, 9.3°). These distortions are described by highly anisotropic AMFs (*u*) along the three basis vectors where the maximum amplitude are along *b* for the carbon atoms of biphenyl (Fig. 2 ▸ and Table S4). Notably, the rotations in the present structure are significantly larger than those reported for molecular biphenyl [φ ≃  ±  5.5° (Petricek *et al.*, 1985 ▸; Baudour & Sanquer, 1983 ▸)]. These values are smaller than those in the low-temperature superstructure of *p*-terphenyl and *p*-quarterphenyl [maximum φ_terphenyl, quarterphenyl_ ≃ 23° (Rice *et al.*, 2013 ▸; Baudour *et al.*, 1976 ▸, 1978 ▸)].

The nature of structural changes in the present system and molecular biphenyl below the phase transition temperature is different to *p*-(*n* > 2)-phenyl systems described by the property that in the later cases two disordered conformations of the molecules freeze by superstructure formation and breaking the monoclinic symmetry of their high-temperature phase.

A distinctive property of the present modulated structure is the unequal modulation for the two different moieties where *u*_biphenyl_ > *u*_phenylalaninate_ (Table S4). Compared to 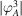 , the distortions in torsion angles φ^1^ are lesser and those in ψ are very small and virtually equal for both *A* and *B* [ 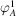 = −133.7°, −127.6°; 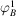 = 55°, 56.2°; 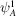 = 36.8°, 37.7°; 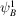 = 36°, 36.6° in Figs. 3 ▸(*c*) and 3 ▸(*d*)].

A possible reason for the weaker modulations of the atoms around the chiral centers is the directional strong intermolecular N—H⋯O bonds makes large intramolecular rotations unfavorable.

Note that the observed changes in the rotations of φ^2^ of molecule *A* [compare 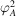 = 39.1°, 25.8° with 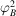 = 32.9°, 28.9° in Fig. 3 ▸(*b*)] are predominantly described by strong modulations of the molecule’s biphenyl moiety. The asymmetry in rotations of individual molecules (Δ|φ^3^| ≈ 5°) is determined by the disparate bonding environments of the biphenyl moieties where the inner rings are that are covalently bonded to amide groups while the outer interact weakly *via* C—H⋯H—C interactions with the phenyl rings of phenylalaninate groups (Fig. 4 ▸). Subsequently, the unequal values at the relevant *t*-sections of φ^3^ are correlated with those of φ^2^ [compare Figs. 3 ▸(*a*) and 3 ▸(*b*)].

It is also observed that the variation in φ^1^ is greater for molecule *A* than that of *B* [compare 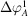 ≃ 6° with 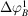 ≃ 1° in Fig. 3 ▸(*c*)]. The origin of disparate distortions in intramolecular rotations is explained in §3.2.

In the modulated structure, the biphenyl moieties in (*AA*)_*n*_ and (*BB*)_*n*_ stacks which are parallel (θ = 0°) in phase I are tilted with respect to each other [Fig. 3 ▸(*e*)]. These tilts (θ_*AA*/*BB*_) are of the order of the internal twists (φ^3^) of the independent biphenyl moieties [θ_*AA*_ = 19.5° and 16.6°; θ_*BB*_ = 5° and 7.2° for inner and outer rings of biphenyl respectively, compare Fig. 3 ▸(*e*) with Fig. 3 ▸(*a*)] The orientation between the biphenyl moieties within the (*ABAB*)_*n*_ stacks also vary with Δθ_*AA*/*BB*_ ≃ 12° where the value is intermediate to 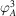 and 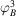 [compare Fig. 3 ▸(*e*) with Fig. 3 ▸(*a*)]. In addition, intermolecular distances between the biphenyl moieties within the stacks at the two *t*-sections are different and vary up to Δ*d*_*AA*/*BB*_ ≃ 0.05 Å and Δ*d*_*ABAB*_ ≃ 0.02 Å [Fig. 3 ▸(*f*)].

Overall distortions in *d*_*AA*_ and *d*_*BB*_ are nearly equal although θ_*AA*_ is greater than θ_*BB*_. These variations in *d* may arise to compensate for the mutual rotations of aromatic rings within the stacks. For example, the comparison θ_*AA*_ for outer rings > θ_*AA*_ for inner rings as opposed to θ_*BB*_ for outer rings < θ_*BB*_ for inner rings could explain the complimentary variations of *d*_*AA*_ and of *d*_*BB*_ [sets of distances (Å) in *t* = ( 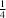 , 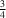 ): *d*_*AA*_ = (5.01, 5.06); *d*_*BB*_ = (5.06, 5.02) in Fig. 3 ▸(*f*)]. It could therefore be argued that the dimerization of biphenyl molecular stacks below *T*_c_ is predominantly governed by distortion described by molecular rotations rather than intermolecular distances. On the other hand, variation of intermolecular distances between aromatic rings of l-phenylalaninate are similar to those of the biphenyls albeit the interstack rotations of the former are significantly smaller (θ < 3°, Fig. S5 in supporting information).

### 3.2. Competitive forces governing modulations

Structural studies in the 3D phase of molecular biphenyl have suggested that the *ortho*-hydrogen atoms are displaced away in the plane of the rings to minimize steric hindrance (Trotter, 1961 ▸; Hargreaves & Rizvi, 1962 ▸; Charbonneau & Delugeard, 1976 ▸). On the other hand, dynamic disorder predominantly governed by torsional vibrations around the long molecular axis (Petricek *et al.*, 1985 ▸) is predicted to balance the planar conformation of biphenyl favorable for crystal packing (Lenstra *et al.*, 1994 ▸).

As short as 1.98 Å in phase I (Table 2 ▸), these contacts are shorter than the predicted values for twice van der Waals radius for hydrogen [*r* = 1.1–1.2 Å (Rowland & Taylor, 1996 ▸; Alvarez, 2013 ▸)]. In the modulated structure, we observe that the distances between the *ortho*-hydrogen atoms are marginally but consistently larger than those in phase I (Table 2 ▸) that could suggest that the torsional modulations aid in minimization of the presumed steric hindrance below *T*_c_ (Dey *et al.*, 2022 ▸, 2018 ▸).

A peculiar property of the modulated structure under discussion is the significant difference in the torsional amplitude φ^3^ of the independent molecules. This aspect cannot be explained solely based on the intramolecular steric factors. Analysis of the crystal packing shows that each of these independent biphenyl moieties maintains close intermolecular CH⋯HC contacts with the phenyl rings of l-phenylalaninate in *AB* and *BA* fashion (Fig. 4 ▸).

These distances are significantly longer (intermolecular *d*_H⋯H_ ≥ 2.4 Å, Table 2 ▸) compared with the intramolecular H⋯H distances. On the other hand, the aromatic rings of l-phenylalaninate interact with adjacent oxygen atoms of –COOCH_3_ *via* C—H⋯O hydrogen bonds (Fig. 4 ▸ and Table 2 ▸). These hydrogen bonds are weaker (Desiraju & Steiner, 2001 ▸) but highly directional [∠(C—H⋯O) = 159–164°] with very little variation in the distances.

Interestingly, those H⋯H distances involving biphenyl moieties of molecule *B* are consistently smaller than those of molecule *A* in both phases (Table 2 ▸). We argue that in the presence of both the van der Waals interactions and weak C—H⋯O bonds, the larger distortions of *A* is favored by weaker CH⋯HC interactions while that is suppressed in *B*.

The variations in φ^2^ and asymmetry in φ^3^ can be explained with respect to the intramolecular nonbonded nearest distances between the hydrogen atoms bonded to C13 atoms (Fig. 1 ▸) of inner rings of biphenyl and the oxygen atoms O3 of the amide groups. The distances are short (*d*_H13⋯O3_ = 2.59 Å in phase I, Table 2 ▸) and are in the range of the sum of the van der Waals radii of oxygen and hydrogens [*r*_H_ = 1.1–1.2 Å, *r*_O_ = 1.4–1.56 Å (Rowland & Taylor, 1996 ▸; Alvarez, 2013 ▸)]. In the modulated structure, the larger distortions of 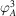 requires the amide groups to rotate with respect to the inner phenyl ring to optimize these C—H⋯O=C contacts to avoid steric effects (compare *d*_H13*a*⋯O3*a*_ = 2.66 Å, 2.54 Å in Table 2 ▸ with 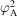 = 39.1°, 25.8° in Fig. 3 ▸). Although the variation in 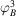 is smaller, the positive correlation that is larger rotations with greater H⋯O distances and *vice versa* are observed (compare *d*_H13*b*⋯O3*b*_ = 2.61 Å, 2.56 Å in Table 2 ▸ with 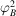 = 32.9°, 28.9° in Fig. 3 ▸). Alternatively, it could be argued that the asymmetric distortion of φ^3^ within individual molecules is a result of the constraint of the amide groups, where rotations are hindered when the C—H⋯O=C contact distances are short and favored when those are longer. Therefore, the four different values of intramolecular torsion φ^3^ within the biphenyl moieties are distinctively governed by intramolecular nonbonded H⋯H and H⋯O; and intermolecular nonbonded H⋯H contacts and weak C—H⋯O hydrogen bonds.

The independent molecules *A* and *B* differ from each other with respect to their torsion around the chiral center namely φ^1^ (= −130.1° and 56.1° for *A* and *B*, respectively, in phase I). The difference in twist angles has remarkable effects on the molecular conformation with respect to the distance between the O3 atoms of the amide groups and hydrogen atoms of the chiral centers C3 carbon atoms (*d*_H3⋯O3_ = 2.37 Å, 3.65 Å for *A* and *B*, respectively, in phase I, Table 2 ▸). In phase II, the modulations of l-phenylalaninate moieties are weaker than that of the biphenyl groups possibly due to the presence of strong intermolecular N—H⋯O hydrogen bonds. However, the variations in 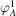 ( ≃6°) as compared with 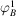 ( ≃1°) are greater which could arise to optimize significantly shorter H3⋯O3 nonbonded contacts of *A* as compared with *B* (*d*_H3⋯O3_ = 2.38 Å, and 3.65 Å for *A* and *B*, respectively, in phase II, Table 2 ▸).

**Figure 5:**
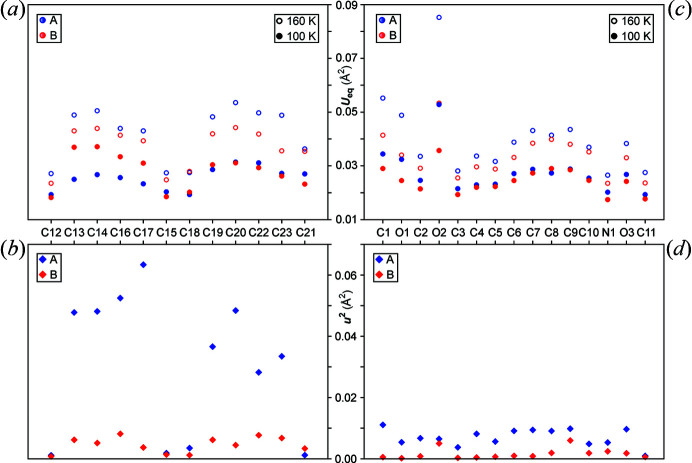
Scatter plots of equivalent value of anisotropic ADPs (*U*_eq_) and square of the amplitude of modulations (*u* ^2^) of the carbon atoms of biphenyl moieties (C12 through to C23) and non-hydrogen atoms of l-phenylalaninate moieties [see Fig. 1 ▸(*a*)] of molecules *A* (blue) and *B* (red). (*a*) and (*c*) *U*_*eq*_ of the biphenyl carbon atoms and l-phenylalaninate non-hydrogen atoms, respectively, at *T* = 160 K (open circles) and at *T* = 100 K (full circles). (*b*) and (*d*) *u* ^2^ (diamonds) of the corresponding biphenyl carbon atoms and l-phenylalaninate non-hydrogen atoms of molecules *A* and *B*, respectively, at *T* = 100 K. See Tables S5 and S6.

As noted in §2.2 the average intensities of the satellite reflections are an order of magnitude smaller than the main reflections. The order parameter of the phase transition might be expressed as proportional to the amplitude of the modulation which is approximately proportional to square root of the intensities of the satellite reflections (van Smaalen, 2005 ▸). The pronounced AMFs of the biphenyl moieties describing the predominant distortions in the crystal structure are accompanied by suppression of dynamic disorder in phase II as compared with phase I. For example, the carbon atoms at *ortho* (C14, C16, C19, C23) and *meta* (C13, C17, C20, C22) positions are strongly displaced [Fig. 5 ▸(*b*), Tables S4 and S5]. Subsequently, the ADPs are significantly reduced as compared with phase I [Fig. 5 ▸(*a*), Table S5]. Notably, the decrease of the ADPs (*U*_eq_) from *T* = 160 K to *T* = 100 K is larger for those of molecule *A* than those for *B* in conjunction with the fact that overall the ADPs are smaller or similar for the former as opposed to phase I, while the square of the amplitude of modulations (*u* ^2^) are greater for *A* than those for *B* [compare Figs. 5 ▸(*b*) with 5 ▸(*a*)].

The decrease in ADPs of atoms of l-phenylalaninate moieties of *A* are greater than *B* from *T* = 160 K to *T* = 100 K [Fig. 5 ▸(*c*)]. Unlike the biphenyl moieties, such apparent switch-over of the ADPs is not observed where the overall values of *A* are greater than those of *B* in both phases I and II, while the squares of the AMFs in II are larger for *A* than those for *B* [compare Figs. 5 ▸(*d*) with 5 ▸(*c*), Tables S4 and S6]. It is possible that the superstructure formation below the phase transition aids in optimal conformation of the biphenyl moieties by significant distortions of their internal torsion while the modulations in l-phenylalaninate groups compensates for the former.

In hindsight, the observed larger mean-squared displacements of atoms of molecule *A* than *B* in high-*T* phase I possibly arises to optimize the short nonbonded H⋯O distances around the chiral center that is largely minimized in phase II by greater intramolecular distortions of torsional angles in the former than the later. This hypothesis is supported by the small but observable distortion of 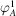 (∼ 6°) as compared with 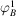 (∼ 1°) while those for ψ are significantly smaller (∼ 1°) for both molecules in phase II.

## 4. Conclusions

The 2*a*  ×  *b*  ×  2*c* superstructure of 4-biphenylcarboxy-(l)-phenylalaninate at *T* = 100 K has been successfully described as a commensurately modulated structure within (3+1)D superspace with superspace group symmetry *P*2_1_(σ_1_0σ_3_)0. The single crystal to single crystal phase transition below *T* = 124 K drives the 3D structure directly to a locked-in twofold superstructure accompanied by significant distortion of torsional rotations within biphenyl away from coplanarity that is also a property of incommensurately modulated structure of biphenyl but with amplitudes four times smaller than the present system.

The phase transition temperature is significantly higher than that in biphenyl yet significantly lower than for *p*-terphenyl and *p*-quarterphenyl. Consistent with the *T*_c_, the maximum amplitude of torsion is also intermediate and in the order φ_quarterphenyl_ ≥ φ_terphenyl_ > φ_4-biphenylcarboxy-l-phenylalaninate_ > φ_biphenyl_.

Topologically separated, conformations of both the weaker C—H⋯O bonds and stronger N—H⋯O bonds are rigid and that underlines their role in stabilizing the crystal packing in both phases. A unique property of the present polyphenyl coupled amino acid ester is the distinctively unequal torsional amplitude (φ_*A*_ > φ_*B*_) within the independent molecules which is governed by multiple level of competitions involving unequal van der Waals constraints in the presence of weak hydrogen bonds between the biphenyl and l-phenylalaninate moieties while the asymmetry of φ^3^ is determined by intramolecular nonbonded constraints between phenyl rings and amide groups.

The unusual nature of the phase transition is rationalised by the fact that unequal intramolecular distortions of the two molecules are complemented by unequal suppression of the dynamic disorder of their atoms below *T*_c_.

The present investigation of the phase transition in the biphenylcarboxy coupled amino acid ester system also shows consequences for crystal packing where significant distortions in conjugations represented by mutual intramolecular rotations between homo aromatic groups as well as with aliphatic amide groups results in modulated π⋯π stacking arrangements (θ_*AA/BB*_ > 0°) of phenyl groups but preserves the conformations of intermolecular directional N—H⋯O hydrogen bonds of the high-temperature structure.

## 5. Related literature

The following references are cited in the supporting information: Becker & Coppens (1974 ▸), Coelho (2003 ▸, 2018 ▸), Petříček *et al.* (2014 ▸). 

## Supplementary Material

Crystal structure: contains datablock(s) global, I_phaseII_modulated, I_phaseI, I_phaseII. DOI: 10.1107/S2052520623000215/bm5150sup1.cif

Structure factors: contains datablock(s) I_phaseII_modulated. DOI: 10.1107/S2052520623000215/bm5150I_phaseII_modulatedsup2.hkl

Structure factors: contains datablock(s) I_phaseI. DOI: 10.1107/S2052520623000215/bm5150I_phaseIsup3.hkl

Structure factors: contains datablock(s) I_phaseII. DOI: 10.1107/S2052520623000215/bm5150I_phaseIIsup4.hkl

Details of structure refinements, powder X-ray di raction experiments, Figs S1-S8, Table S1-S8. DOI: 10.1107/S2052520623000215/bm5150sup5.pdf

0YDiRh5cfxe

CCDC references: 2235161, 2238250, 2238251

## Figures and Tables

**Table 1:** Temperature dependence of unit-cell parameters and the components of the modulation wavevector, σ_1_ and σ_3_ See Table S1 in supporting information for reflections used.

*T* (K)	*a* (Å)	*b* (Å)	*c* (Å)	β (°)	σ_1_	σ_3_	*V* (Å^3^)
200†	5.0560 (3)	8.6622 (4)	42.242 (3)	90.349 (4)			1850.00 (18)
160	5.0479 (2)	8.6330 (4)	42.1525 (15)	90.513 (3)			1836.87 (13)
150	5.0498 (7)	8.6161 (8)	42.136 (11)	90.607 (11)			1833.2 (5)
140	5.0484 (6)	8.6093 (7)	42.145 (10)	90.661 (10)			1831.6 (5)
130	5.0451 (7)	8.6014 (8)	42.120 (11)	90.713 (11)			1827.6 (6)
128	5.0446 (7)	8.6002 (8)	42.113 (11)	90.723 (11)			1826.9 (6)
126	5.0440 (6)	8.5992 (7)	42.114 (10)	90.720 (9)			1826.5 (5)
124	5.0443 (6)	8.5978 (7)	42.100 (10)	90.733 (9)	0.499 (9)	0.51 (7)	1825.7 (5)
122	5.0440 (7)	8.5970 (7)	42.100 (10)	90.745 (10)	0.498 (6)	0.49 (4)	1825.5 (5)
120	5.0433 (7)	8.5984 (7)	42.100 (10)	90.746 (10)	0.497 (5)	0.52 (3)	1825.5 (5)
118	5.0441 (7)	8.5958 (8)	42.092 (11)	90.764 (10)	0.500 (4)	0.50 (3)	1824.8 (5)
116	5.0432 (6)	8.5926 (7)	42.087 (9)	90.775 (9)	0.500 (4)	0.51 (3)	1823.6 (5)
114	5.0422 (6)	8.5923 (7)	42.090 (10)	90.787 (10)	0.500 (4)	0.53 (3)	1823.3 (5)
100	5.0377 (2)	8.5898 (3)	42.0432 (14)	90.884 (3)	0.5	0.5	1819.11 (11)

† Sasmal *et al.* (2019*a* ▸).

**Table 2:** Comparison of nonbonded hydrogen⋯acceptor and hydrogen⋯hydrogen distances (Å) involved in hydrogen bonds and steric factors in phase I (*T*_1_) and phase II (*T*_2_) The two values for distances in phase II (*T*_2_) correspond to *t* = 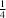 and *t* = 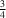 , respectively.

Interaction class	Atom labels	Phase	Distance (Å)
N—H⋯O	H1n1a⋯O3a^i^	I	2.00
		II	1.97, 1.95
	H1n1b⋯O3b^ii^	I	2.00
		II	2.00, 1.98
C—H⋯O	H1c10a⋯O2a^iii^	I	2.72
		II	2.72, 2.68
	H1c10b⋯O2b^iv^	I	2.78
		II	2.74, 2.73
Intra H⋯H	H1c14a⋯H1c19a	I	1.98
		II	2.08, 2.09
	H1c14b⋯H1c19b	I	1.98
		II	2.00, 1.99
	H1c16a⋯H1c23a	I	2.03
		II	2.10, 2.16
	H1c16b⋯H1c23b	I	2.02
		II	2.03, 2.06
Intra H⋯O	H1C13a⋯O3a	I	2.59
		II	2.66, 2.54
	H1C13b⋯O3b	I	2.59
		II	2.61, 2.56
Intra H⋯O	H1C3a⋯O3a	I	2.37
		II	2.38, 2.38
	H1C3b⋯O3b	I	3.65
		II	3.65, 3.65
Inter H⋯H	H1c21a⋯H1c7b	I	2.59
		II	2.61, 2.56
	H1c21b⋯H1c7a^ii^	I	2.45
		II	2.39, 2.46

Symmetry codes. Phase I: (i) *x* − 1, *y*, *z*; (ii) *x* + 1, *y*, *z*; (iii) 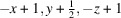 ; (iv) 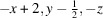 . Phase II: (i) *x* − 1, *y*, *z*, *t*; (ii) *x*+ 1, *y*, *z*, *t*; (iii) 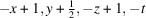 ; (iv) 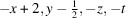 .

## References

[bb2] Alvarez, S. (2013). *Dalton Trans.* **42**, 8617–8636. 10.1039/c3dt50599e 23632803

[bb3] Bastiansen, O. (1949). *Acta Chem. Scand.* **3**, 408–414.

[bb4] Baudour, J. L., Delugeard, Y. & Cailleau, H. (1976). *Acta Cryst.* B**32**, 150–154.

[bb5] Baudour, J.-L., Délugeard, Y. & Rivet, P. (1978). *Acta Cryst.* B**34**, 625–628.

[bb6] Baudour, J. L. & Sanquer, M. (1983). *Acta Cryst.* B**39**, 75–84.

[bb102] Becker, P. J. & Coppens, P. (1974). *Acta Cryst.* A**30**, 129–147.

[bb7] Benkert, C. & Heine, V. (1987). *Phys. Rev. Lett.* **58**, 2232–2234. 10.1103/PhysRevLett.58.2232 10034687

[bb8] Benkert, C., Heine, V. & Simmons, E. H. (1987). *J. Phys. C Solid State Phys.* **20**, 3337–3354.

[bb9] Bree, A. & Edelson, M. (1977). *Chem. Phys. Lett.* **46**, 500–504.

[bb10] Bree, A. & Edelson, M. (1978). *Chem. Phys. Lett.* **55**, 319–322.

[bb11] Bürkle, M., Viljas, J. K., Vonlanthen, D., Mishchenko, A., Schön, G., Mayor, M., Wandlowski, T. & Pauly, F. (2012). *Phys. Rev. B*, **85**, 075417.

[bb12] Busing, W. R. (1983). *Acta Cryst.* A**39**, 340–347.

[bb13] Cailleau, H., Moussa, F. & Mons, J. (1979). *Solid State Commun.* **31**, 521–524.

[bb14] Casalone, G., Mariani, C., Mugnoli, A. & Simonetta, M. (1968). *Mol. Phys.* **15**, 339–348.

[bb15] Chapuis, G. (2020). *Acta Cryst.* B**76**, 510–511. 10.1107/S205252062000949X 32831268

[bb16] Charbonneau, G.-P. & Delugeard, Y. (1976). *Acta Cryst.* B**32**, 1420–1423.

[bb17] Charbonneau, G. P. & Delugeard, Y. (1977). *Acta Cryst.* B**33**, 1586–1588.

[bb100] Coelho, A. A. (2003). *J. Appl. Cryst.* **36**, 86–95.

[bb101] Coelho, A. A. (2018). *J. Appl. Cryst.* **51**, 210–218.

[bb18] Cranney, M., Comtet, G., Dujardin, G., Kim, J. W., Kampen, T. U., Horn, K., Mamatkulov, M., Stauffer, L. & Sonnet, P. (2007). *Phys. Rev. B*, **76**, 075324.

[bb19] Desiraju, G. R. & Steiner, T. (2001). *The Weak Hydrogen Bond. In Structural Chemistry and Biology*, 1st ed. Oxford University Press.

[bb20] Dey, S., Schönleber, A., Mondal, S., Ali, S. I. & van Smaalen, S. (2018). *Cryst. Growth Des.* **18**, 1394–1400.

[bb21] Dey, S., Schönleber, A., Mondal, S., Prathapa, S. J., van Smaalen, S. & Larsen, F. K. (2016). *Acta Cryst.* B**72**, 372–380. 10.1107/S2052520616005503 PMC4886617 27240768

[bb22] Dey, S., Schönleber, A., van Smaalen, S., Morgenroth, W. & Krebs Larsen, F. (2022). *Chem. Eur. J.* **28**, e202104151. 10.1002/chem.202104151 PMC9303887 35072296

[bb23] Ecolivet, C., Sanquer, M., Pellegrin, J. & DeWitte, J. (1983). *J. Chem. Phys.* **78**, 6317–6324.

[bb24] Friedman, P. S., Kopelman, R. & Prasad, P. N. (1974). *Chem. Phys. Lett.* **24**, 15–17.

[bb25] Hargreaves, A. & Rizvi, S. H. (1962). *Acta Cryst.* **15**, 365–373.

[bb26] Hochstrasser, R. M., McAlpine, R. D. & Whiteman, J. D. (1973). *J. Chem. Phys.* **58**, 5078–5088.

[bb27] Ishibashi, Y. (1981). *J. Phys. Soc. Jpn*, **50**, 1255–1258.

[bb28] Janner, A. & Janssen, T. (1977). *Phys. Rev. B*, **15**, 643–658.

[bb29] Janssen, T., Chapuis, G. & de Boissieu, M. (2018). *Aperiodic Crystals: from Modulated Phases to Quasicrystals: Structure and Properties*, 2nd ed. Oxford University Press.

[bb30] Jeong, H., Li, H. B., Domulevicz, L. & Hihath, J. (2020). *Adv. Funct. Mater.* **30**, 2000615.

[bb57] Johansson Seechurn, C. C. C., Kitching, M. O., Colacot, T. J. & Snieckus, V. (2012). *Angew. Chem. Int. Ed.* **51**, 5062–5085. 10.1002/anie.201107017 22573393

[bb31] Khatua, R., Sahoo, S. R., Sharma, S. & Sahu, S. (2020). *Synth. Met.* **267**, 116474.

[bb32] Lenstra, A. T. H., Van Alsenoy, C., Verhulst, K. & Geise, H. J. (1994). *Acta Cryst.* B**50**, 96–106.

[bb33] Mishchenko, A., Vonlanthen, D., Meded, V., Bürkle, M., Li, C., Pobelov, I. V., Bagrets, A., Viljas, J. K., Pauly, F., Evers, F., Mayor, M. & Wandlowski, T. (2010). *Nano Lett.* **10**, 156–163. 10.1021/nl903084b 20025266

[bb34] Nespolo, M. (2019). *Acta Cryst.* A**75**, 551–573. 10.1107/S2053273319000664 31041910

[bb35] Noohinejad, L., Mondal, S., Ali, S. I., Dey, S., van Smaalen, S. & Schönleber, A. (2015). *Acta Cryst.* B**71**, 228–234. 10.1107/S2052520615004084 PMC4383393 25827376

[bb36] Oniwa, K., Kanagasekaran, T., Jin, T., Akhtaruzzaman, M., Yamamoto, Y., Tamura, H., Hamada, I., Shimotani, H., Asao, N., Ikeda, S. & Tanigaki, K. (2013). *J. Mater. Chem. C*, **1**, 4163–4170.

[bb37] Palatinus, L. & Chapuis, G. (2007). *J. Appl. Cryst.* **40**, 786–790.

[bb38] Parlinski, K., Schranz, W. & Kabelka, H. (1989). *Phys. Rev. B*, **39**, 488–494. 10.1103/physrevb.39.488 9947178

[bb39] Petricek, V., Coppens, P. & Becker, P. (1985). *Acta Cryst.* A**41**, 478–483.

[bb40] Petříček, V., Dušek, M. & Palatinus, L. (2014). *Z. Kristallogr. Cryst. Mater.* **229**, 345–352.

[bb41] Petříček, V., Dušek, M. & Plášil, J. (2016). *Z. Kristallogr. Cryst. Mater.* **231**, 583–599.

[bb43] Pinheiro, C. B. & Abakumov, A. M. (2015). *IUCrJ*, **2**, 137–154. 10.1107/S2052252514023550 PMC4285887 25610634

[bb44] Popelier, P. L. A., Maxwell, P. I., Thacker, J. C. R. & Alkorta, I. (2019). *Theor. Chem. Acc.* **138**, 12. 10.1007/s00214-018-2383-0 PMC6383956 30872951

[bb45] Ramakrishnan, S., Schönleber, A., Hübschle, C. B., Eisele, C., Schaller, A. M., Rekis, T., Bui, N. H. A., Feulner, F., van Smaalen, S., Bag, B., Ramakrishnan, S., Tolkiehn, M. & Paulmann, C. (2019). *Phys. Rev. B*, **99**, 195140.

[bb46] Rekis, T., Schönleber, A., Noohinejad, L., Tolkiehn, M., Paulmann, C. & van Smaalen, S. (2021). *Cryst. Growth Des.* **21**, 2324–2331.

[bb47] Rekis, T., Schönleber, A. & van Smaalen, S. (2020). *Acta Cryst.* B**76**, 18–27. 10.1107/S2052520619014938 PMC8788847 32831236

[bb48] Rice, A. P., Tham, F. S. & Chronister, E. L. (2013). *J. Chem. Crystallogr.* **43**, 14–25.

[bb1] Rigaku Oxford Diffraction (2019). *CrysAlisPro.* Rigaku Corporation, Oxford, UK.

[bb49] Rowland, R. S. & Taylor, R. (1996). *J. Phys. Chem.* **100**, 7384–7391.

[bb50] Saito, K., Atake, T. & Chihara, H. (1985). *J. Chem. Thermodyn.* **17**, 539–548.

[bb51] Sasmal, S., Nandi, S. K., Kumar, S. & Haldar, D. (2019*a*). *ChemistrySelect*, **4**, 11172–11176.

[bb52] Sasmal, S., Podder, D., Debnath, M., Nandi, S. K. & Haldar, D. (2019*b*). *ChemistrySelect*, **4**, 10302–10306.

[bb53] Schoenleber, A. (2011). *Z. Kristallogr.* **226**, 499–517.

[bb54] Schönleber, A. & Chapuis, G. (2004). *Acta Cryst.* B**60**, 108–120. 10.1107/S0108768103027605 14734850

[bb55] Schönleber, A., Meyer, M. & Chapuis, G. (2001). *J. Appl. Cryst.* **34**, 777–779.

[bb56] Schönleber, A., Pattison, P. & Chapuis, G. (2003). *Z. Kristallogr.* **218**, 507–513.

[bb58] Smaalen, S. van (2005). *Acta Cryst.* A**61**, 51–61.

[bb59] Smaalen, S. van (2012). *Incommensurate Crystallography*, 1st ed. Oxford University Press.

[bb60] Smaalen, S. van, Campbell, B. J. & Stokes, H. T. (2013). *Acta Cryst.* A**69**, 75–90. 10.1107/S0108767312041657 PMC3553647 23250064

[bb61] Steed, K. M. & Steed, J. W. (2015). *Chem. Rev.* **115**, 2895–2933. 10.1021/cr500564z 25675105

[bb62] Stokes, H. T., Campbell, B. J. & van Smaalen, S. (2011). *Acta Cryst.* A**67**, 45–55. 10.1107/S0108767310042297 21173472

[bb63] Suzuki, H. (1959). *Bull. Chem. Soc. Jpn*, **32**, 1340–1350.

[bb64] Trotter, J. (1961). *Acta Cryst.* **14**, 1135–1140.

[bb65] Vonlanthen, D., Mishchenko, A., Elbing, M., Neuburger, M., Wandlowski, T. & Mayor, M. (2009). *Angew. Chem. Int. Ed.* **48**, 8886–8890. 10.1002/anie.200903946 19847835

[bb66] Wagner, T. & Schönleber, A. (2009). *Acta Cryst.* B**65**, 249–268. 10.1107/S0108768109015614 19461136

[bb67] Wakayama, N. I. (1981). *Chem. Phys. Lett.* **83**, 413–417.

[bb68] Wei, J., Liang, B., Duan, R., Cheng, Z., Li, C., Zhou, T., Yi, Y. & Wang, Y. (2016). *Angew. Chem. Int. Ed.* **55**, 15589–15593. 10.1002/anie.201607653 27862811

[bb69] Wolff, P. M. de (1974). *Acta Cryst.* A**30**, 777–785.

[bb70] Yamamura, Y., Saito, K., Ikemoto, I. & Sorai, M. (1998). *J. Phys. Condens. Matter*, **10**, 3359–3366.

