# Supplementary material for: Asymmetric rotations and dimerization driven by normal to modulated phase transition in 4-biphenylcarboxy coupled l-phenylalaninate
Source: Acta Crystallogr B Struct Sci Cryst Eng Mater. 2023 Mar 2;79(Pt 2):148–56. doi: 10.1107/S2052520623000215 (PMC10088479; doi:10.1107/S2052520623000215)
Supplement: Supplementary file 5 [file b-79-00148-sup5.pdf]

Acta Cryst B

STRUCTURAL SCIENCE
CRYSTAL ENGINEERING
MATERIALS

Volume 79 (2023)

Supporting information for article:

Asymmetric rotations and dimerization driven by normal to modulated phase transition in 4-biphenylcarboxy coupled l-phenylalaninate

Somnath Dey, Supriya Sasmal, Saikat Mondal, Santosh Kumar, Rituparno Chowdhury, Debashrita Sarkar, C. Malla Reddy, Lars Peters, Georg Roth and Debasish Haldar

Supporting information

Asymmetric rotations and dimerization driven by normal to modulated phase transition in 4-biphenylcarboxy coupled L-phenylalaninate

Somnath Dey[a,b*], Supriya Sasmal[a], Saikat Mondal[a], Santosh Kumar[a], Rituparno Chowdhury[a], Debashrita Sarkar[a], C. Malla Reddy[a], Lars Peters[b], Georg Roth[b] and Debasish Haldar[a]

[a]*Department of Chemical Sciences, Indian Institute of Science Education and Research (IISER) Kolkata, Mohanpur 741246, India,*

[b]*Institute of Crystallography, RWTH Aachen University, Jägerstraße 17-19, 52066 Aachen, Germany*

E-mail: somnathdey226@gmail.com

Contents

Details of structure refinements

Powder X-ray diffraction experiments

Supplementary Figures S1–S8

Supplementary Table S1–S8.

Details of structure refinements of different modulated structure models

Structure refinements have been performed using JANA2006[1] and JANA2020[2]. Structural model at T = 160 K has been used as an initial model for the basic structure of the modulated structure at T = 100 K. All atoms were set to isotropic for displacement parameters and the model was refined against main reflections [$R_F^{obs}(m{=}0)$ = 0.0723]. In the next step, first order harmonic for displacive modulation was described for all atoms and an incommensurate (IC) model was refined against main and satellite reflections. Refinement led to improved fit to the main reflections [$R_F^{obs}(m{=}0)$ = 0.0586, $R_F^{obs}(m{=}1)$ = 0.1215]. Refinement of the anisotropic atomic displacement parameters (ADPs) of all non-hydrogen atoms resulted in significant improvement to the residual values [IC model A: $R_F^{obs}(m{=}0)$ = 0.0374, $R_F^{obs}(m{=}1)$ = 0.0771] and residual features ($\Delta\rho_{min}/\Delta\rho_{max}$) decreased from -0.68/1.23 eÅ^{-3} to -0.33/0.31 eÅ^{-3}. However, ADPs of four non-hydrogen atoms were found to be non-positive definite. Further test by describing first order harmonic for ADP modulation for all non-hydrogen atoms model led to improvement of the residual values [IC model A: $R_F^{obs}(m{=}0)$ = 0.0363, $R_F^{obs}(m{=}1)$ = 0.0677] but ADPs of 11 non-hydrogen atoms were found to be non-positive definite along certain t-sections. This model was discarded for further analysis.

In the next step, IC model A was used as a starting model to describe three commensurate (C) models by fixing the initial phase of the modulation, $t_0 = 0$, $\frac{1}{4}$ and $\frac{1}{8}$ respectively. The former two t_0 values correspond to $2a \times b \times 2c$ superstructure in 3D with monoclinic symmetry $B2_1$ while the later correspond to a superstructure with triclinic $B1$ symmetry. Restrictions on t_0 values also impose constraints on the refinable variables corresponding to atomic modulation functions (AMFs). These restrictions follow the argument that the total number of refinable parameters in the equivalent 3D superstructure and their (3+1)D commensurately modulated structural models must be equal. In the present case, either sin or cos waves can be refined for structural models with $t_0 = 0$ and $\frac{1}{4}$ because the point group symmetry is same in their corresponding 3D superstructure models. On the other hand, assumed monoclinic to triclinic distortion in the 3D superstructure (space group $B1$)corresponding to (3+1)D C model with $t_0 = \frac{1}{8}$ can be derived by using both components of the Fourier series. It must be noted that such restrictions on sin and cos waves cannot be formally imposed on the AMFs of hydrogen atoms in JANA2006 and JANA2020 as their modulations are fully determined by geometrical conditions of the riding model. $t_0 = \frac{1}{4}$ yielded the best fit to the diffraction data (Table S3) with reduced number of parameters as compared to the IC model A (compare $N_{C,t_0=0.25} = 649$ to $N_{IC,model\,A} = 811$). Most importantly, ADPs of all non-hydrogen atoms are positive definite.

Notably, the residual values of the IC model as well as the C model at $t_0 = \frac{1}{8}$ is marginally

smaller than for the C model at $t_0 = \frac{1}{4}$. Assuming all the three models should fit similarly to the diffraction data for equivalent descriptions of structural models further tests included attempts to refine the IC model and C model at $t_0 = \frac{1}{8}$ with reduced number of parameters (= 649) similar to $t_0 = \frac{1}{4}$. Refinements led to worse fit with large R-values (Table S3).

In the final step, all reflections were averaged in monoclinic symmetry corresponding to $t_0 = \frac{1}{4}$. One parameter corresponding to isotropic extinction correction was refined. Finally, fractional co-ordinates and AMFs for hydrogen atoms belonging to N–H groups involved in strong hydrogen bonds improved the fit to the diffraction data marginally (R_F^{obs} = 0.0419 in Table S2).

Additional refinement was performed including first order harmonic for anisotropic ADPs of all non-hydrogen atoms. Refinement of this model with additional 324 parameters converged with marginal improvement of R_F^{obs} (= 0.0406) values. However, the residual density $\Delta\rho_{min}/\Delta\rho_{max}$ remained unchanged [compare -0.26/0.28 e/Å^3 to -0.25/0.29 e/Å^3] and 306 parameters refined to values within three times their standard uncertainties. The model was therefore discarded. Thus the superspace approach reduced the total numbers of refinable parameters by $\sim$ 33 %.

Additional X-ray diffraction experiments

Powder X-ray diffraction experiments were performed on thoroughly ground powder of the compound at ambient conditions using a Rigaku SmartLab with a CuKα radiation. JANA2006 was used to index the diffraction patterns. For reference, lattice parameters at ambient conditions were obtained from single crystal X-ray diffraction (SCXRD) experiment at ambient conditions (Table S8). The PXRD pattern could not be indexed using the lattice parameters as obtained from the SCXRD data [Fig. S8(a)] that suggest that the compound undergoes phase transition upon grinding. Lattice parameters were calculated employing the singular value decomposition (SVD)-Index algorithm in TOPAS[3;4]. The PXRD pattern could be indexed using a primitve triclinic cell (Cell 1) with unit cell volume comparable to that of single crystal [Table S8, Fig S8(b)]. Another triclinic cell (Cell 2) could also describe the pattern [Fig. S8(c)]. Le Baile refinements of the patterns against both the cells resulted in similar residual values (Table S8). However, Cell 1 fits better to the PXRD than Cell 2 [compare inset plots of Fig S8(b) and Fig S8(c)]. In addition, the unit cell volume of Cell 2 is larger than 7.5 % to that of the single crystal that implies different density of the ground material.

Based on this difference of phases between single crystals (monoclinic structure) and pulverised material (triclinic structure), T-dependent PXRD experiments to complement the single crystal to single crystal phase transition in this material was not pursued.

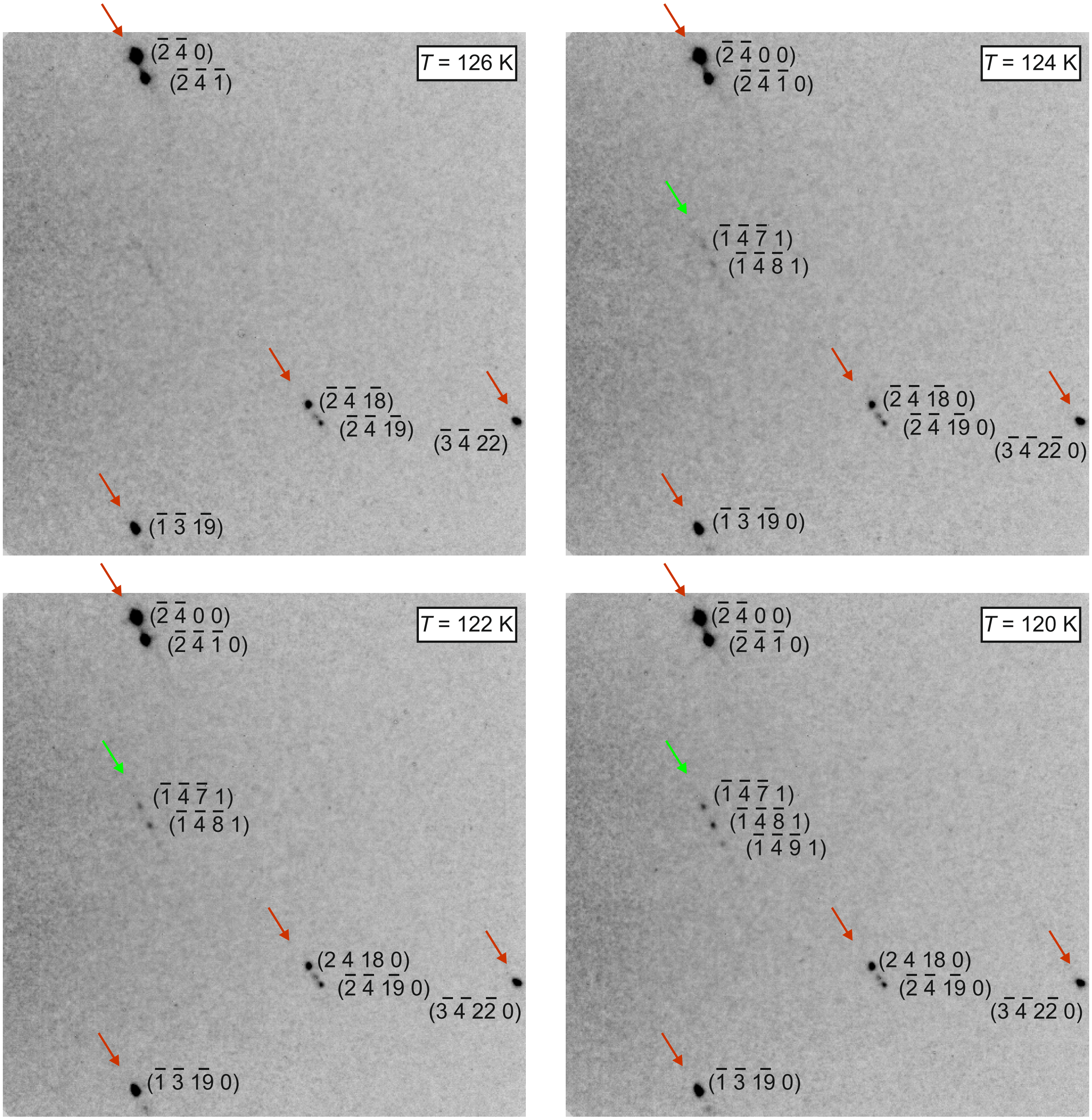

Figure S1: Diffraction images across the normal (phase I) to commensurately modulated (phase II) phase transition. Red arrows depict Bragg peaks in phase I and main Bragg peaks in phase II. The satellites are diffuse at $T = 124$ K (green arrow) that becomes stronger at lower temperatures. Reflections at $T = 126$ K are indexed using three integers (hkl) and at $T_c = 124$ K and lower temperatures by four integers $(hklm)$, where $m = 0$ and $m = 1$ for main and satellite reflections respectively. Image resolution range in $d \sim 2.2$ to 1.1 Å.

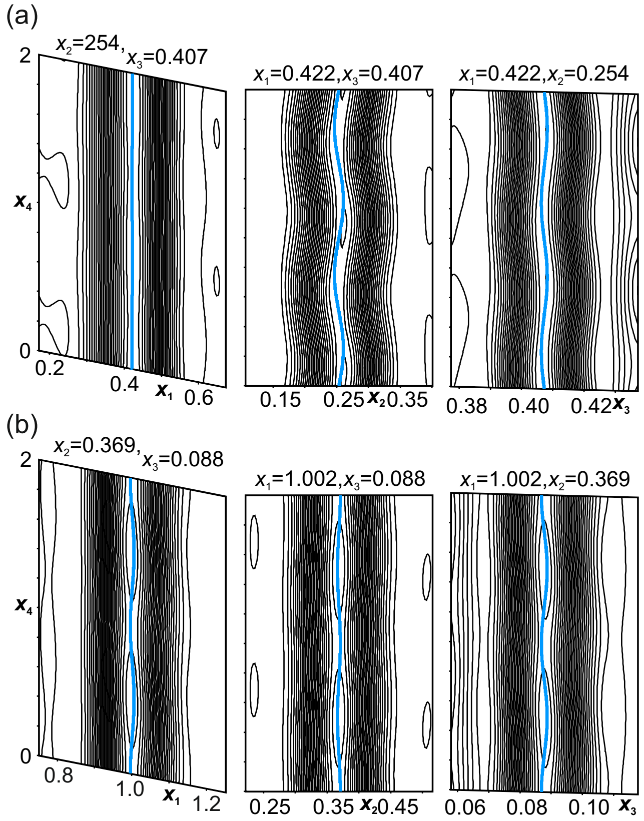

Figure S2: (x_{si},x_{s4})-sections of Fourier map centered on nitrogen atoms (light blue) of amide groups (a) atom N1a of molecule 'A' and (b) atom N1b of molecule 'B'. The contour line and the width of the maps are 0.5 eÅ^{-3} and 2.5 Å respectively.

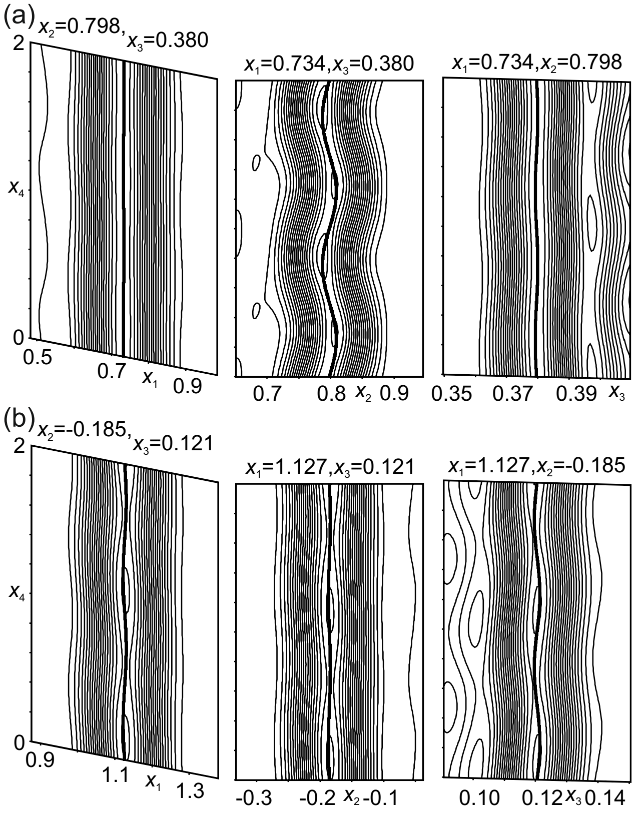

Figure S3: (x_{si},x_{s4})-sections of Fourier map centered on carbon atoms (black) (a) C8a of molecule 'A' and (b) C8b of molecule 'B' respectively belonging to the phenyl ring of L-phenylalaninate moieties. The contour line and the width of the maps are 0.5 eÅ^{-3} and 2.5 Å respectively.

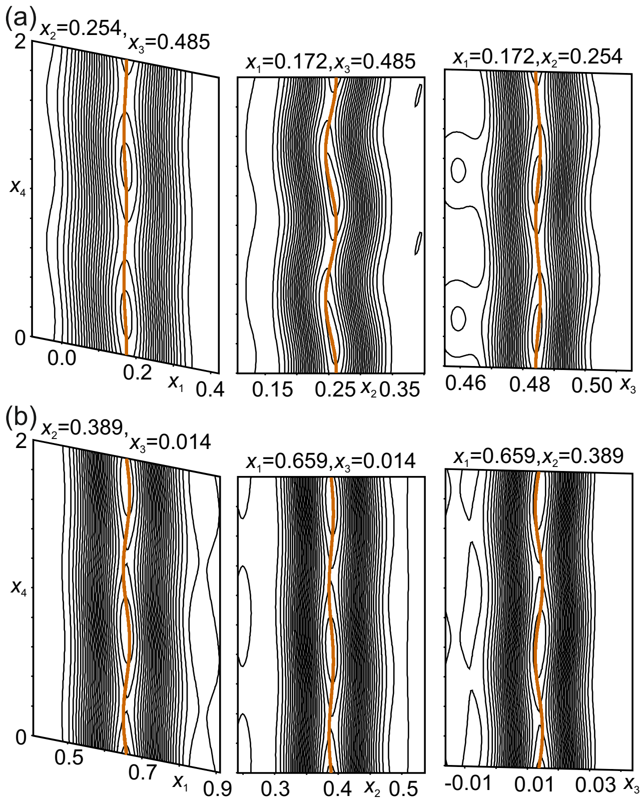

Figure S4: (a) and (b) (x_{si},x_{s4})-sections of Fourier map centered on oxygen atoms (orange) O2a of molecule 'A' and O2b of molecule 'B' respectively belonging to carboxylate groups of L-phenylalaninate moieties. The contour line and the width of the maps are 0.5 eÅ^{-3} and 2.5 Å respectively.

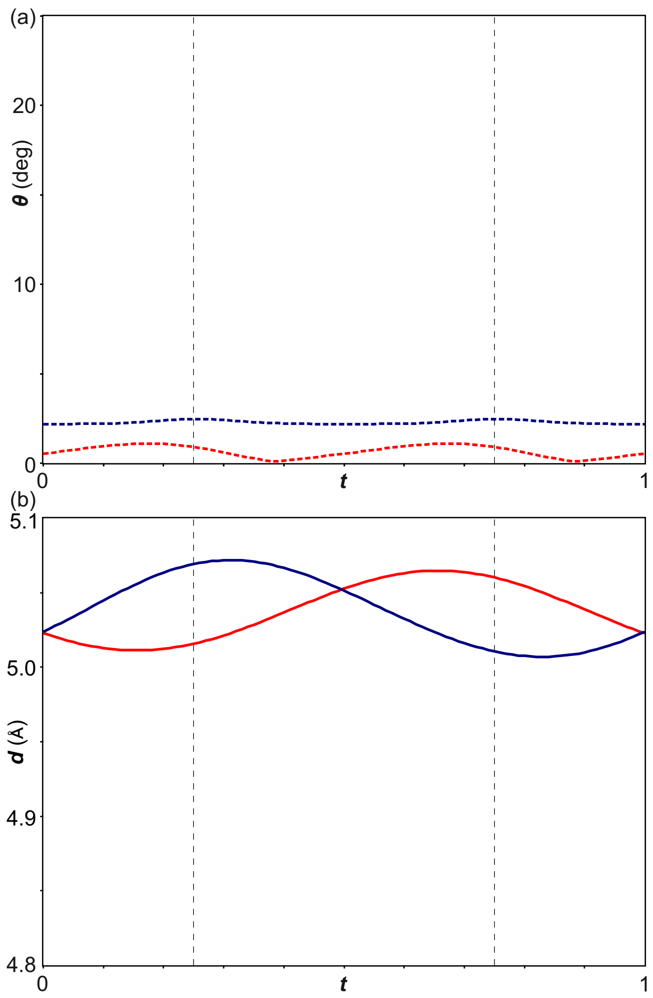

Figure S5: t-plots of (a) angle (θ) and (b) distances (d). θ and d represent tilt and intermolecular distance respectively between phenyl rings of L-phenylalaninate moieties of 'A' and 'A'^{ii} (blue) and between those of 'B' and 'B'^{ii} (red). Symmetry code:(ii) $x+1, y, z, t$.

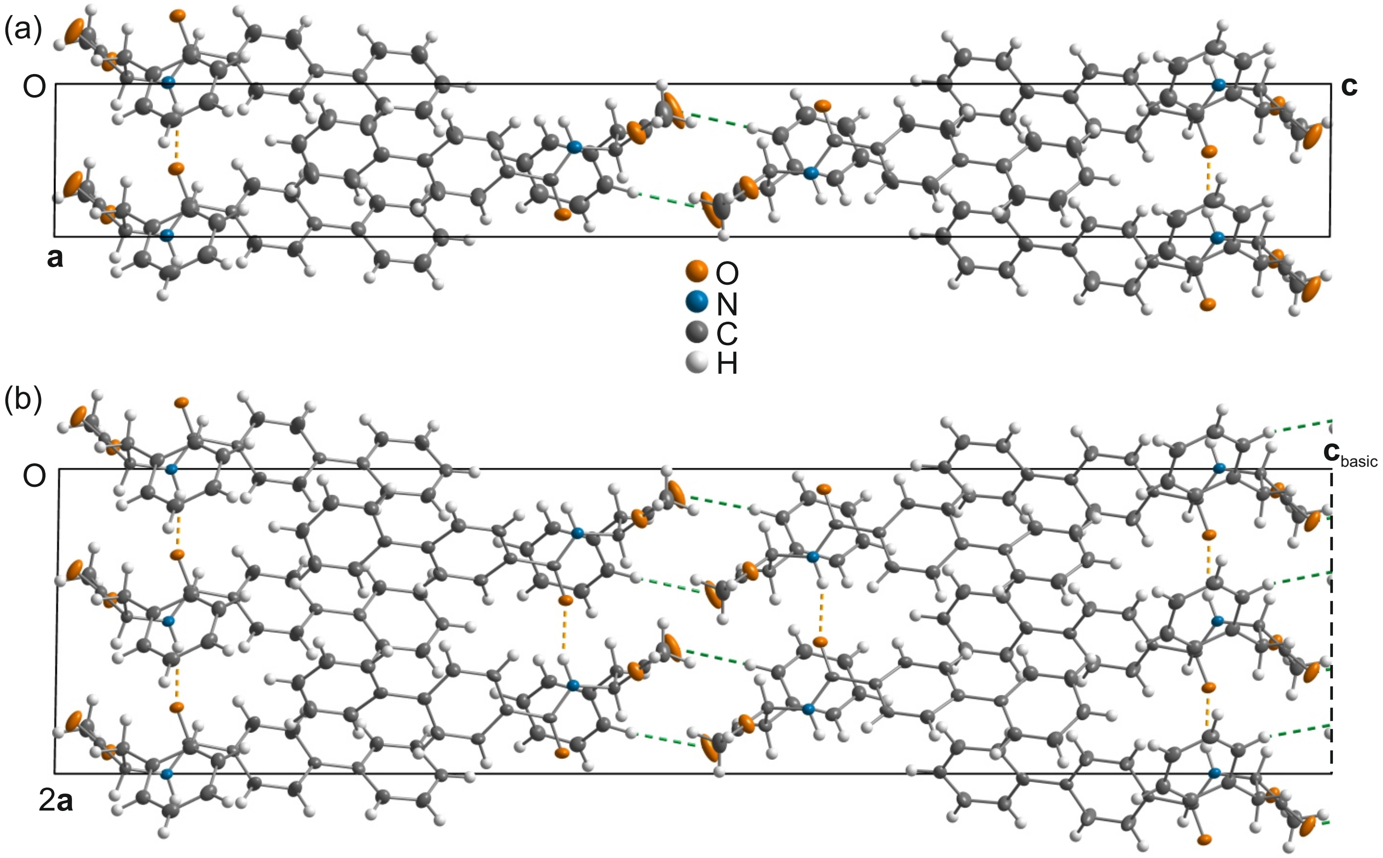

Figure S6: Crystal packing of 4-biphenylcarboxy-(L)-phenylalaninate at (a) T = 160 K (phase I) and (b) Section of the $2a \times b \times 2c$ superstructure at T = 100 K (phase II) drawn up to $c_{\text{basic}} = \frac{1}{2}c_{\text{superstructure}}$. Dashed orange lines depict linear N–H···O hydrogen bonds along [∓100] directions, while green dashed lines represent C–H···O hydrogen bond dimers. Displacement ellipsoids are cut at 50% probability level. Viewing direction along [010].

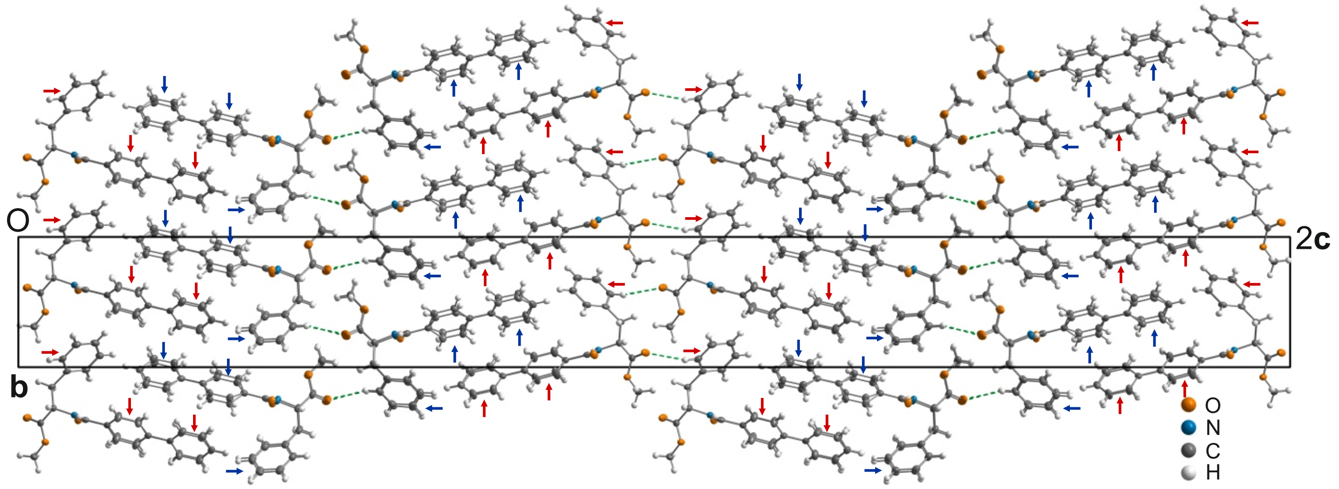

Figure S7: View along **a** of the $2a \times b \times 2c$ superstructure at $T = 100$ K (phase II) illustrating the dimerization of the molecular stacks along the [100] direction. Tilts (θ in Fig. 3 of article) between adjacent aromatic rings of biphenyl groups of 'A' and between those of 'B' in $(AA)_n$ and $(BB)_n$ stacks are indicated by blue and red vertical arrows respectively; and θ (Fig. S5) between phenyl rings of L-phenylalaninate moieties in $(AA)_n$ and $(BB)_n$ stacks are indicated by blue and red horizontal arrows respectively. Green dashed lines represent C–H···O hydrogen bonds. Displacement ellipsoids are cut at 50% probability level.

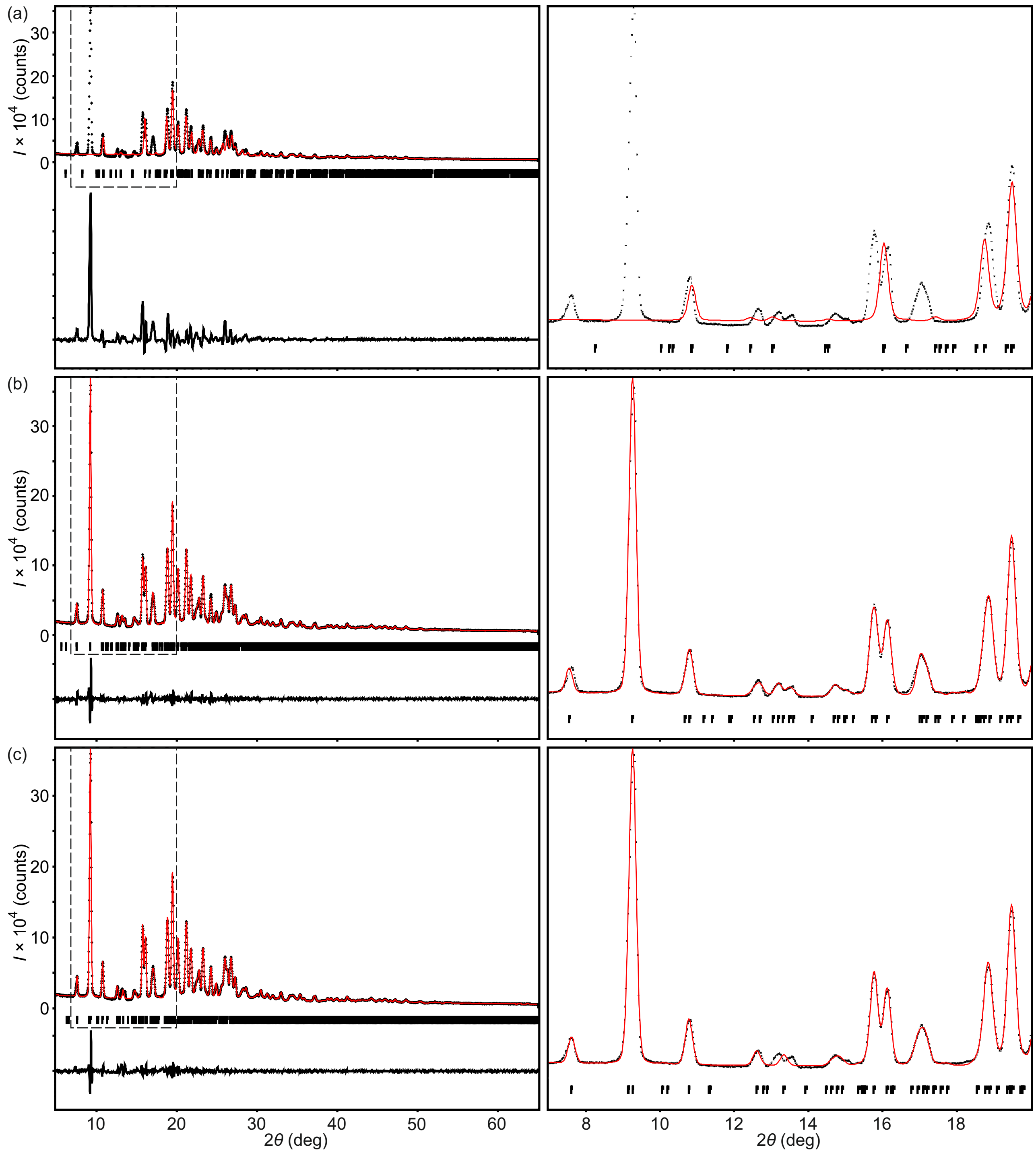

Figure S8: Comparison of fit of the experimental powder X-ray diffraction pattern to (a) as obtained unit cell from SCXRD, (b) Calculated unit cell 1 with volume 1901 Å^3 and (c) calculated unit cell with volume 2029 Å^3. Experimental pattern, calculated profile and difference are given in black cross points, red curve and black curve respectively. The insets in 2θ = 7-20 deg are given in the right column corresponding to the area (dashed rectrangle) in left.

Table S1: Technical details of SCXRD measurements and number of reflections used for calculation of lattice parameters and components of modulation wave vector, $\mathbf{q}$.

T (K)	Number of runs	Number of images	d_{min} (Å)	Number of reflections
160	27	1345	0.84	3437
150	9	45	0.84	130
140	9	45	0.84	140
130	9	45	0.84	136
128	9	45	0.84	134
126	9	45	0.84	139
124	9	45	0.84	135
122	9	45	0.84	147
120	9	45	0.84	152
118	9	45	0.84	149
116	9	45	0.84	159
114	9	45	0.84	161
100	26	1486	0.84	3948

Table S2: Experimental and crystallographic data

Crystal data		
Chemical formula	$C_{23}H_{21}NO_3$	
M_r	359.42	
Temperature (K)	160	100
Crystal system	Monoclinic b-unique	Monoclinic b-unique
a, b, c (Å)	5.0479(2), 8.6330(4), 42.1525(15)	5.0377(2), 8.5898(3), 42.0432(14)
β (deg)	90.513(3)	90.884(3)
V (Å^3)	1836.87(13)	1819.11(11)
Wave vector ($\mathbf{q}$)	–	$\frac{1}{2}\mathbf{a}^* + \frac{1}{2}\mathbf{c}^*$
Space group	$P2_1$	–
Superspace group	–	$P2_1(\sigma_1 0 \sigma_3)0$
Commensurate section	–	$t_0 = \frac{1}{4}$
Supercell	–	$2a{\times}b{\times}2c$
Supercell space group	–	$B2_1$
Diffraction data		
Wavelength	CuKα	
d (Å)	0.84	0.84
$\Delta\omega$ (deg)	1	1
Absorption correction	multiscan	
Criterion of observability	$I > 3\sigma(I)$	
Unique reflections		
all (obs/all)	4219/4555	5940/8898
$m = 0$ (obs/all)	–	4150/4390
$m = 1$ (obs/all)	–	1790/4508
R_{int} (obs/all)	0.0200/0.0202	0.0248/0.0274
GoF (obs/all)	1.57/1.54	1.60/1.40
$R_F^{obs}/wR_{F^2}^{all}$		
all (obs/all)	0.0393/0.0492	0.0419/0.0526
$m = 0$ (obs/all)	–	0.0368/0.0460
$m = 1$ (obs/all)	–	0.0791/0.1191
$\Delta\rho_{min}/\Delta\rho_{max}$ (e/Å^3)	-0.15/0.17	-0.26/0.28
No. of parameters	494	662
H-atom treatment	mixed	mixed
Extinction correction	Isotropic type I[5]	
Extinction coefficient	0.34(3)	0.20(3)
Twin matrix	100 0-10 00-1	
Twin volumes	0.9760(8)/0.0240(8)	0.9758(7)/0.0242(7)

Table S3: Statistical parameters (R_F^{obs}, $wR_{F^2}^{all}$) of the (3+1)D incommensurately modulated (IC) and commensurately modulated (C) refinements of models with different values of the phase t_0. Number of reflections (obs/all) used in the refinements are averaged for the lowest triclinic point group symmetry: (m=0) = 4911/5241, (m=11) = 2031/5537. Space group (SG) symmetries of the equivalent 3D superstructures corresponding to different C structures are given which for the IC structure is meaningless.

	IC		$t_0 = 0$	$t_0 = \frac{1}{8}$		$t_0 = \frac{1}{4}$
SG	–		$B2_1$	$B1$		$B2_1$
No. of parameters	811	649	649	811	649	649
GoF(obs/all)	1.55/1.35	2.93/2.56	2.68/2.38	1.56/1.33	2.04/1.81	1.55/1.35
(R_F^{obs}(all)	0.0428	0.0745	0.0691	0.0428	0.0566	0.0433
$wR_{F^2}^{all}$ (all)	0.0539	0.1035	0.0961	0.0533	0.0731	0.0547
$R_F^{obs}(m = 0)$	0.0381	0.0424	0.0401	0.0384	0.0406	0.0384
$wR_{F^2}^{all}$ $(m = 0)$	0.0473	0.0515	0.0492	0.0475	0.0498	0.0476
$R_F^{obs}(m = 1)$	0.0777	0.3119	0.2838	0.0758	0.1750	0.0800
$wR_{F^2}^{all}$ $(m = 1)$	0.1232	0.3174	0.3653	0.1166	0.2402	0.1280
$\Delta\rho_{min}/\Delta\rho_{max}$ (e/Å^3)	-0.33/0.31	-1.28/1.30	-1.04/1.09	-0.31/0.30	-0.79/0.84	-0.36/0.30
-ve ADPs	4	4	1	none	2	none
correlations > 0.6	1	20	1	98	247	1

Table S4: Components of the amplitude of atomic modulation functions ($|u_x|$, $|u_y|$ and $|u_z|$) along the three basis vectors **a**, **b** and **c** respectively for molecules A and B.

Atom	$|u_x|$ (Å)		$|u_y|$ (Å)		$|u_z|$ (Å)	
	A	B	A	B	A	B
C1	0.0015	0.0086	0.0996	0.0198	0.0340	0.0092
O1	0.0070	0.0050	0.0670	0.0120	0.0294	0.0071
C2	0.0156	0.0166	0.0739	0.0129	0.0319	0.0198
O2	0.0181	0.0433	0.0721	0.0283	0.0311	0.0483
C3	0.0121	0.0081	0.0498	0.0077	0.0336	0.0134
C4	0.0408	0.0045	0.0730	0.0112	0.0340	0.0172
C5	0.0348	0.0070	0.0610	0.0077	0.0265	0.0244
C6	0.0242	0.0010	0.0876	0.0009	0.0290	0.0311
C7	0.0045	0.0066	0.0945	0.0002	0.0219	0.0286
C8	0.0040	0.0196	0.0936	0.0112	0.0172	0.0378
C9	0.0217	0.0388	0.0910	0.0361	0.0328	0.0563
C10	0.0337	0.0136	0.0515	0.0198	0.0328	0.0357
N1	0.0030	0.0247	0.0610	0.0180	0.0399	0.0387
O3	0.0055	0.0045	0.0936	0.0249	0.0294	0.0345
C11	0.0025	0.0035	0.0129	0.0155	0.0282	0.0189
C12	0.0171	0.0141	0.0137	0.0120	0.0256	0.0210
C13	0.1083	0.0171	0.1898	0.0636	0.0029	0.0433
C14	0.1098	0.0146	0.1898	0.0584	0.0027	0.0391
C15	0.0191	0.0257	0.0266	0.0180	0.0277	0.0202
C16	0.1501	0.0821	0.1623	0.0352	0.0597	0.0130
C17	0.1657	0.0539	0.1787	0.0283	0.0631	0.0029
C18	0.0237	0.0247	0.0275	0.0103	0.0467	0.0219
C19	0.1264	0.0640	0.1366	0.0455	0.0446	0.0042
C20	0.1446	0.0423	0.1580	0.0507	0.0500	0.0105
C21	0.0081	0.0332	0.0060	0.0258	0.0332	0.0399
C22	0.0972	0.0463	0.1349	0.0567	0.0244	0.0483
C23	0.0922	0.0348	0.1572	0.0618	0.0160	0.0416

Table S5: Equivalent value of the ADP tensors, (U_{eq}) of atoms of the biphenyl moieties at T = 160 K (phase I), T = 100 K (phase II) and their differences (ΔU_{eq}); and the sum of the square of the amplitudes of their atomic modulation functions along three basis vectors (u^2) for molecules A and B . $u^2 = (u_x)^2 + (u_y)^2 + (u_z)^2$.

Atom label	Molecule	$U_{eq,PhaseI}$ (Å^2)	$U_{eq,PhaseII}$ (Å^2)	ΔU_{eq} (Å^2)	u^2 (Å^2)
C12	A	0.0271	0.0193	0.0078	0.0011
	B	0.0235	0.0182	0.0053	0.0008
C13	A	0.0489	0.0250	0.0239	0.0478
	B	0.0430	0.0369	0.0061	0.0062
C14	A	0.0505	0.0267	0.0238	0.0481
	B	0.0439	0.0371	0.0068	0.0051
C15	A	0.0274	0.0203	0.0071	0.0018
	B	0.0248	0.0185	0.0063	0.0014
C16	A	0.0439	0.0256	0.0183	0.0525
	B	0.0414	0.0334	0.0080	0.0081
C17	A	0.0430	0.0233	0.0197	0.0634
	B	0.0393	0.0310	0.0083	0.0037
C18	A	0.0275	0.0193	0.0082	0.0035
	B	0.0279	0.0202	0.0077	0.0012
C19	A	0.0482	0.0286	0.0196	0.0366
	B	0.0419	0.0304	0.0115	0.0062
C20	A	0.0535	0.0314	0.0221	0.0484
	B	0.0442	0.0311	0.0131	0.0045
C21	A	0.0363	0.0270	0.0093	0.0012
	B	0.0354	0.0232	0.0122	0.0034
C22	A	0.0497	0.0311	0.0186	0.0282
	B	0.0418	0.0293	0.0125	0.0077
C23	A	0.0488	0.0272	0.0216	0.0335
	B	0.0356	0.0262	0.0094	0.0068

Table S6: Equivalent value of the ADP tensors, (U_{eq}) of atoms of the L-phenylalaninate moieties at $T = 160$ K (phase I), $T = 100$ K (phase II) and their differences (ΔU_{eq}); and the sum of the square of the amplitudes of their atomic modulation functions along three basis vectors (u^2) for molecules A and B . $u^2 = (u_x)^2 + (u_y)^2 + (u_z)^2$.

Atom label	Molecule	$U_{eq,PhaseI}$ (Å^2)	$U_{eq,PhaseII}$	ΔU_{eq} (Å^2)	u^2 (Å^2)
C1	A	0.0552	0.0344	0.0208	0.0111
	B	0.0414	0.0290	0.0124	0.0005
O1	A	0.0488	0.0324	0.0164	0.0054
	B	0.0340	0.0245	0.0095	0.0002
C2	A	0.0335	0.0246	0.0089	0.0067
	B	0.0291	0.0214	0.0077	0.0008
O2	A	0.0852	0.0528	0.0314	0.0065
	B	0.0533	0.0357	0.0176	0.0050
C3	A	0.0281	0.0215	0.0066	0.0038
	B	0.0255	0.0193	0.0062	0.0003
C4	A	0.0336	0.0229	0.0107	0.0082
	B	0.0296	0.0220	0.0076	0.0004
C5	A	0.0316	0.0232	0.0084	0.0056
	B	0.0288	0.0223	0.0065	0.0007
C6	A	0.0388	0.0271	0.0117	0.0091
	B	0.0331	0.0245	0.0086	0.0010
C7	A	0.0431	0.0287	0.0144	0.0094
	B	0.0384	0.0273	0.0111	0.0009
C8	A	0.0414	0.0273	0.0141	0.0091
	B	0.0398	0.0290	0.0108	0.0019
C9	A	0.0435	0.0288	0.0147	0.0098
	B	0.0380	0.0285	0.0095	0.0060
C10	A	0.0369	0.0254	0.0115	0.0049
	B	0.0352	0.0246	0.0106	0.0018
N1	A	0.0265	0.0202	0.0063	0.0053
	B	0.0235	0.0174	0.0061	0.0024
C11	A	0.0275	0.0193	0.0082	0.0010
	B	0.0236	0.0177	0.0059	0.0006
O3	A	0.0383	0.0268	0.0115	0.0096
	B	0.0330	0.0242	0.0138	0.0018

Table S7: Comparison of interatomic bond distances (Å) of molecules A and B in phase I (T = 160 K) and phase II (T = 100 K).

	phase I		phase II			
Atom groups	A	B	A		B	
			$t = \frac{1}{4}$	$t = \frac{3}{4}$	$t = \frac{1}{4}$	$t = \frac{3}{4}$
C1–O1	1.45	1.45	1.44	1.45	1.45	1.45
O1–C2	1.31	1.33	1.32	1.32	1.33	1.33
C2–O2	1.18	1.20	1.20	1.19	1.20	1.21
C2–C3	1.52	1.53	1.52	1.52	1.52	1.52
C3–C4	1.53	1.55	1.54	1.54	1.55	1.56
C4–C5	1.51	1.51	1.50	1.51	1.51	1.51
C5–C6	1.39	1.40	1.38	1.39	1.39	1.40
C6–C7	1.39	1.38	1.38	1.38	1.38	1.38
C7–C8	1.37	1.38	1.39	1.40	1.38	1.38
C8–C9	1.38	1.39	1.38	1.38	1.40	1.39
C9–C10	1.39	1.40	1.39	1.39	1.38	1.38
C10–C5	1.39	1.38	1.40	1.39	1.39	1.39
C3–N1	1.45	1.45	1.45	1.46	1.45	1.45
N1–C11	1.34	1.33	1.33	1.33	1.32	1.33
C11–O3	1.23	1.23	1.24	1.24	1.24	1.23
C11–C12	1.50	1.50	1.50	1.50	1.50	1.51
C12–C13	1.37	1.37	1.39	1.38	1.37	1.37
C13–C14	1.39	1.39	1.39	1.38	1.39	1.39
C14–C15	1.38	1.38	1.40	1.39	1.39	1.39
C15–C16	1.38	1.37	1.40	1.38	1.38	1.38
C16–C17	1.39	1.39	1.39	1.39	1.38	1.39
C17–C12	1.37	1.38	1.39	1.38	1.39	1.38
C15–C18	1.49	1.50	1.48	1.49	1.50	1.50
C18–C19	1.39	1.40	1.39	1.40	1.39	1.39
C19–C20	1.39	1.38	1.38	1.38	1.39	1.38
C20–C21	1.36	1.38	1.39	1.39	1.37	1.38
C21–C22	1.36	1.37	1.38	1.37	1.37	1.37
C22–C23	1.39	1.38	1.38	1.39	1.37	1.39
C23–C18	1.38	1.39	1.39	1.40	1.40	1.39

Table S8: Comparison of lattice parameters and residual values from Le baile fit of the PXRD pattern based on two unit cells. Lattice parameters obtained from SCXRD data has been given as reference.

	SCXRD	PXRD	
		Cell 1	Cell 2
a (Å)	5.0646(2)	14.5357(12)	13.7041(10)
b (Å)	8.7483(3)	8.6153(6)	13.0856(9)
c (Å)	42.4157(15)	16.5541(12)	11.4692(8)
α (deg)	90	108.248(5)	89.337(7)
β (deg)	90	103.902(4)	99.307(5)
γ (deg)	90	80.233(5)	89.436(7)
V (Å^3)	1879.27(12)	1901.1(3)	2029.4(3)
GoF	–	3.51	3.53
R_p/wR_p	–	0.0422/0.0642	0.0416/0.0646

References

[1] V. Petricek, M. Dusek, and L. Palatinus, Z. Kristallogr. **229**, 345 (2014).

[2] V. Petricek et al, JANA2020, Institute of Physics, Prague, Czech Republic (2022).

[3] A. A. Coelho, J. Appl Crystallogr. **51**, 210 (2018).

[4] A. A. Coelho, J. Appl Crystallogr. **36**, 86 (2003).

[5] P. J. Becker and P. Coppens, Acta Crystallogr. A **30**, 129 (1974).
